# A Model for the Interplay of Receptor Recycling and Receptor-Mediated Contact in T Cells

**DOI:** 10.1371/journal.pone.0000633

**Published:** 2007-07-25

**Authors:** Sergey N. Arkhipov, Ivan V. Maly

**Affiliations:** Department of Computational Biology, University of Pittsburgh School of Medicine, Pittsburgh, Pennsylvania, United States of America; Birmingham University, United Kingdom

## Abstract

Orientation of organelles inside T cells (TC) toward antigen-presenting cells (APC) ensures that the immune response is properly directed, but the orientation mechanisms remain largely unknown. Structural dynamics of TC are coupled to dynamics of T-cell receptor (TCR), which recognizes antigen on the APC surface. Engagement of the TCR triggers its internalization followed by delayed polarized recycling to the plasma membrane through the submembrane recycling compartment (RC), which organelle shares intracellular location with the TC effector apparatus. TCR engagement also triggers TC-APC interface expansion enabling further receptor engagement. To analyze the interplay of the cell-cell contact and receptor dynamics, we constructed a new numerical model. The new model displays the experimentally observed selective stabilization of the contact initiated next to the RC, and only transient formation of contact diametrically opposed to the RC. In the general case wherein the TC-APC contact is initiated in an arbitrary orientation to the RC, the modeling predicts that the contact dynamics and receptor recycling can interact, resulting effectively in migration of the contact to the TC surface domain adjacent to the submembrane RC. Using three-dimensional live-cell confocal microscopy, we obtain data consistent with this unexpected behavior. We conclude that a TC can stabilize its contact with an APC by aligning it with the polarized intracellular traffic of TCR. The results also suggest that the orientation of TC organelles, such as the RC and the effector apparatus, toward the APC can be achieved without any intracellular translocation of the organelles.

## Introduction

It is becoming increasingly recognized that cellular processes, such as those underlying the immune response, may involve non-intuitive interactions between diverse sub-processes and system components, and that our understanding of the system-level effects can be greatly enhanced by employing numerical computer models. The model presented here was designed to predict the dynamics of receptor-mediated immunological cell interactions from experimentally measured kinetic parameters and to be tested against structural cell dynamics observed in experiments. Building on the previous efforts in the field of immunological kinetic modeling, introduction of new dynamic variables for the first time made possible prediction of the relative stability and localization of the immunological synapse, which are important in immunological cell interactions. The chief aim of the reported modeling is quantitatively consistent explanation of experiments conducted previously and design of new experiments, whose results are also presented here.

Pairwise interactions of TC with APC of the immune system and with infected cells are central to the cellular immune response. In different situations, these TC-APC interactions may trigger a variety of specific responses, including activation of TC, induction of immunological memory, lysis (destruction) of the infected and tumorous cells, and production of antibodies [1]. The specificity of these responses is underlain on the molecular level by the specificity of recognition of antigen displayed on the plasma membrane (PM) of the APC by TCR on the PM of the TC. TCR is continuously and actively redistributed in the TC through a cycle of internalization and re-expression on the PM [2]. Recycling is polarized and contributes to accumulation of TCR in the area of the TC-APC interface [3]. Numerical models of recycling explained TCR partitioning between the PM and the intracellular pool [4]. More recently, a model of recycling also addressed the polarized TCR accumulation on the TC-APC interface [5]. However, the interface area in this model was a fixed compartment. In reality, the TCR engagement at the interface triggers expansion of the interface itself and therefore involvement of more of the membrane and receptors in the TC-APC interaction [6], [7]. Here we present a spatially-distributed cell-scale kinetic model that accounts for the interplay between the TCR recycling and the dynamics of the TCR-mediated interface. The model provided a quantitatively consistent explanation for our previous experiments and also exhibited unanticipated behavior that suggested new experiments that are reported here.

The new model is intended to capture a number of features of the TCR-mediated TC-APC interaction in quantitative detail known from experiments. TCR is constitutively internalized from the PM. It is then directed in vesicles along microtubules into the RC [8]. The latter resides, together with the Golgi apparatus (GA), near the point of convergence of the microtubules, which is termed centrosome or microtubule-organizing center (MTOC). The RC-MTOC-GA organelle complex is typically located eccentrically in the TC, next to the PM [3], [5], [9], [10]. TCR is recycled back to the PM adjacent to the RC [3], from where it can diffuse laterally over the entire cell surface [11]. Two effects are triggered when the TC comes in contact with a specific APC, and when TCR on the TC surface recognizes antigen on the surface of the APC. Firstly, the engagement of the receptors triggers expansion of the cell-cell contact area (called immunological synapse) and incorporation of more membrane with receptors into it [6], [7]. Secondly, the internalization of stimulated receptors is sharply accelerated [2], [4]. The outcome of these two effects, which act correspondingly as positive and negative feedback mechanisms, may be either stabilization of the TCR-mediated cell-cell contact or its collapse. Substantial duration of contact is thought to be required for the cell-cell interaction to be effective, e.g. for the TC to deliver the cytotoxins that kill the APC that is infected by viruses. Moreover, the contact needs to be stabilized specifically in the region of the TC surface that is adjacent to the submembrane RC-MTOC-GA complex, because the effector apparatus of the TC is also part of that complex [9], [10], [12], [13]. The numerical model presented here attempts to predict the dynamics of the TCR-mediated TC-APC contact zone from the experimentally measured parameters of TCR recycling for the purpose of comparison with the experimentally observed TCR-mediated contact dynamics.

The new model is a continuous generalization of the compartmentalized model of TCR dynamics [5]. In the previous model, the TC surface, on which TCR is distributed, was treated not as continuous but as divided into three “compartments”, one of which represented to the TC-APC contact zone. This approach made it possible to address the effects of recycling on the dynamics of the receptor number at the TC-APC interface. The compartmentalized model could not, however, account for the receptor-mediated expansion of the interface, which expansion by itself changes the number of the receptors involved in the cell-cell interaction [7]. Some effects of the interface expansion were introduced into that model as receptor flux into the interface compartment from the rest of the PM. Measurements show that most of this apparent lateral convection of TCR reflects its movement with the PM becoming part of the expanding cell-cell contact [7]. Since, however, the interface compartment in the previous numerical model was considered to be of fixed size, the phenomenological receptor influx into it was pre-determined and did not depend on the receptor density already at the interface. The model therefore did not incorporate any positive feedback from receptor engagement that could work against the negative feedback of the accelerated receptor internalization from the interface. In the continuous model presented here, the lateral convection of TCR into the interface area is modeled explicitly as the incorporation of the cell surface into the interface as the latter expands.

To model the TC-APC contact expansion (and retraction), we introduce moving boundaries of the TC-APC contact zone into a continuous model of the TC surface, using some concepts of modeling receptor-mediated adhesion [14]–[18]. Adapting the notion of the critical receptor density required for adhesion from the leukocyte attachment model [14], we model the boundary of the TC-APC contact as advancing if the local TCR density is above some critical value, and as retreating if it is below this value. Overall, the boundary velocity is modeled as a linear function of the local surface TCR density. This simple assumption closes the positive feedback loop between the interfacial receptor density and the involvement of new receptors in the interaction. It reflects the fact that the TCR engagement at the interface stimulates actin-driven expansion of the interface [19]–[21] as well as the more direct impact of the receptor-mediated adhesion into the contact formation [16]. In this regard, our model is an application to the TC and TCR of the concept of receptor-mediated cell adhesion gradients generated by intracellular receptor trafficking [15]–[18]. Although the linear dependence of the boundary velocity on the local TCR density is a crude phenomenological approximation of the details of TCR binding and of the active contact expansion and retraction, we view it as a reasonably mechanistic assumption in a model whose goal is to address the cell-level TCR dynamics in the TC-APC interaction. Taking into account both the recycling and expansion effects on the TCR partitioning into and out of the synapse, the model presented here is suitable for analyzing the dynamic interplay of these two effects. The introduction into the model of the new dynamic variables specifying the location of the boundaries of the interface makes possible explicit prediction of the functionally important stability and localization of the TC-APC contact from the spatially organized kinetics of TCR recycling. Explanation of experiments from this standpoint is the primary goal of this work.

Modeling of the dynamic partitioning of TCR into and out of the synapse is simplified by a possibility to exclude some processes from explicit consideration on the grounds of time-scale and space-scale separation. The receptor residence times in the PM and in the RC are much shorter (minutes) than the lifetime of the receptors before they are biochemically degraded (hours, [2]). This allows considering the total number of receptors in the cell as constant in the model of the effects of recycling during the TC-APC interaction on the time scale of few tens of minutes [2]. Conversely, the intracellular vesicular transport is fast [22], suggesting travel times of the internalized TCR into the RC less than 1 min. This allows treating the traffic *per se* as instantaneous in the model of TCR recycling [5]. Membrane curvature and co-partitioning with other transmembrane molecules were shown to be essential for the finer-scale distribution of TCR within the immunological synapse [23]–[25]. Concerning ourselves here exclusively with the cruder, cell-scale TCR distribution, we omit these effects from our model. Although focusing exclusively on TCR and on the contact dynamics this receptor mediates is justified when modeling our experimental system that engages only this main TC receptor type [19]–[21], the results should only be extrapolated with caution to the real TC-APC interaction that involves many more receptor types [26]. Finally, we make the simplifying assumption of co-modulation [2], [5], considering all TCR within the synapse as subject to the same rapid induced internalization. In reality, only a fraction of the TCR in the synapse may be activated, which has been a subject of intensive theoretical and experimental research [27]–[31]. Our model does not account for these intricate local dynamics of TCR binding, concentrating instead on the cell-scale TCR redistribution.

Our first aim in this work was explaining specific experiments [5] that we had conducted on an experimental model that replaces the APC with an artificial TCR-binding surface [12], [19]–[21]. To this end we first applied the new spatial-kinetic formalism to analyze the kinetic origin of the contact stability in the case where the TC contacts the TCR-binding surface with the side of the cell next to which the intracellular RC is located and to which the polarized recycling is directed. This experimental situation matches the structural polarity in functional and stable TC-APC pairs [3], [9], [10]. Then the new model was applied to the experimental case where the RC remains diametrically opposed to the cell side that is in contact with the TCR-binding substrate. This experimental situation [5] represents the failure of structural polarization in TC [9], [12], which makes the TC-APC interaction nonfunctional [10], [13]. We conducted numerical analysis to determine if the model can reproduce the abortion of contact formation that we observed in this experimental situation and that was TCR recycling-dependent [5]. We then further generalized the modeling by considering initial conditions where the RC is oriented arbitrarily with respect to the contact initiation site. The unexpected numerical results in this case prompted us to re-evaluate some of the assumptions regarding the causality in T cell polarization and to conduct new experiments, which supported the model predictions.

## Results

### The model reproduces the differential contact stability conditioned on the recycling polarity

**Figure 1:**
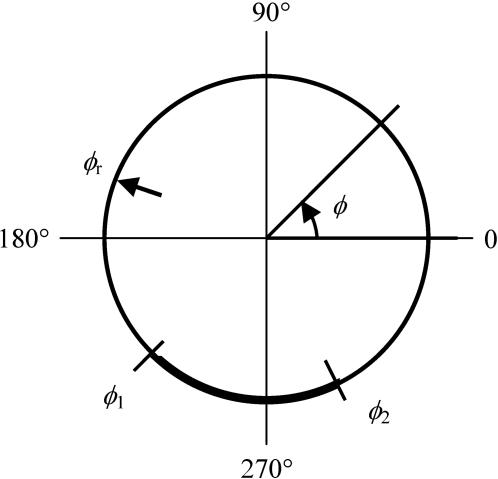
Schematic of the mathematical model. The circumference represents the surface of the TC, on which TCR is distributed by lateral diffusion. Position on the surface is specified by the angular coordinate *Φ* as shown. *Φ*_1_ and *Φ*_2_ denote the instantaneous positions of the two moving boundaries of the TC-APC contact area shown as the heavy arc. TCR is internalized from the contact area with the high ligand-induced rate constant *k*_i_, and from the rest of the surface, with the lower constitutive rate contact *k*_c_. Polarized recycling of the internalized receptors is directed to the position denoted *Φ*_r_, which is adjacent to the eccentric intracellular RC not shown in the diagram. The recycling position and the positions of the boundaries as shown are arbitrary. In the model, the recycling position *Φ*_r_ is fixed and the boundaries *Φ*_1_ and *Φ*_2_ can move along the surface, expanding or contracting the contact area according to whether the local receptor density *P*(*Φ*, *t*) at the boundaries is above or below the critical density *p*_crit_ for attachment to the APC.

The spatial layout of the model is shown diagrammatically in Fig. 1 (see also the complete mathematical formulation in the Materials and Methods section). The model describes redistribution of TCR in a TC interacting with an APC and the dynamics of the TC-APC contact. To this end, the surface of the TC is modeled as a circumference, on which the TCR is distributed, and a part of which represents the area of contact with the APC. A specific position on the surface is referenced by the angular coordinate *Φ*. It is counted between 0 and 360° following the convention of the polar coordinates, so that the top of the cell has *Φ* = 90° and the bottom, where the contact with the TCR-binding surface is invariably initiated in our experimental setup, has the coordinate *Φ* = 270°. The two boundaries that delimit the arc representing the contact area of the TC with the APC (or with the biomimetic TCR-binding surface) can move; their instantaneous positions on the circumference are given by the angular coordinates *Φ*_1_ and *Φ*_2_. The surface TCR distribution is described mathematically by the density function *P*(*Φ*), which denotes the local surface density (concentration) of receptors at the position with the coordinate *Φ*. The time-dependent variable *r* describes the amount of TCR in the intracellular RC. The internalization flux from all of the PM is directed in the model into the RC, and the flux out of the RC (recycling proper) is directed to one fixed point on the PM, whose position is given by *Φ_r_*. This single point is an idealization that represents the cell surface area immediately adjacent to the RC lying under the membrane on the side of the cell, where is recycling is thereby directed. The recycling rate constant is denoted *k*_r_. The surface TCR is subject to lateral diffusion with the diffusion constant *D*. The important aspect of the model is the coupling between the TCR and contact dynamics. Between the two boundaries of the contact area, receptor internalization occurs with the high ligand-induced rate constant *k*_i_, whereas in the rest of the model PM it proceeds with the relatively low constitutive rate constant *k*_c_. The principal novel feature of the model is that the two boundaries are moving laterally on the model cell surface at speeds that are determined by the local TCR density. The simplifying assumption is that the instantaneous boundary speed is a linear function of the local receptor density. The receptor-mediated TC-APC contact formation is thereby captured by two adjustable parameters, the critical receptor density *p*_crit_ and the angular rate constant *k*_ω_ in the following manner. It is assumed that if the local receptor density at the contact boundary it is higher than *p*_crit_, the boundary is advancing so as to expand the contact. If it is lower than *p*_crit_, the boundary is retracting so as to make the contact narrower. Precisely how fast the boundary is advancing or retracting, depending on the deviation of the local receptor density from *p*_crit_, is determined by the rate constant *k*_ω_. The computational details of the model are given in the Materials and Methods section.

In the first model case, we assumed that the RC-MTOC-GA complex was polarized to the area of contact from the very beginning. This situation had been modeled experimentally and the contact stability in it had been measured [5]. In the experimental setup, the contact is initiated at the bottom of the roughly spherical TC. To model the polarity of recycling in this case, we specified the surface point of recycling at the bottom of the cell (*Φ*_r_ = 270°).

The distribution of TCR at the moment of the contact initiation (*t* = 0) should be the steady-state TCR distribution in an isolated TC. This distribution can be obtained as the steady-state solution to a variant of the model without the contact boundaries and with the internalization proceeding at the low constitutive rate on the entire cell surface. In this basal steady state, TCR was predicted to be partitioned 82:18 between the surface and the intracellular pool. This ratio was in very close agreement with the previous non-spatial model [4]. Due to the polarized recycling, TCR was predicted by the present model to be distributed unevenly within the PM in the basal steady state. The TCR surface density was predicted to be 1.5 times higher at the bottom of the cell, near where the recycling was directed, than at the opposite, upper pole of the cell. This degree of surface TCR polarization was in a very close agreement with the previous model that had not been spatially continuous, but distinguished three imaginary regions on the PM [5].

To begin modeling the TCR-mediated contact formation, we introduced the two boundaries of the nascent contact at the bottom of the cell (*Φ*_1_,_2_(0) = 270°). From that point on, the boundaries moved according to the receptor density at their position, and the receptor internalization between the boundaries proceeded at the high ligand-induced rate.

**Figure 2:**
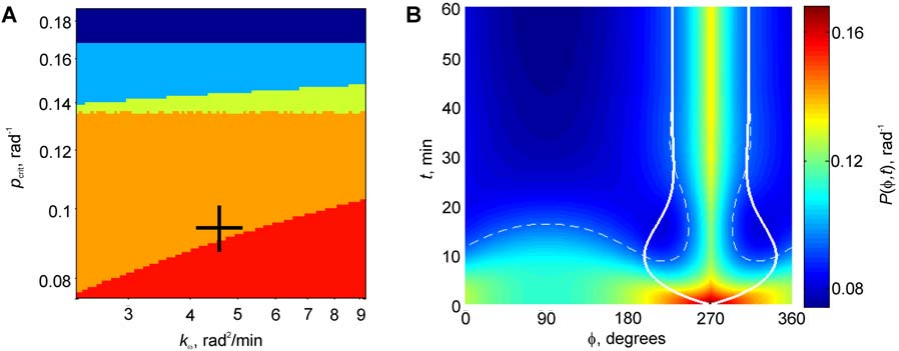
Model behavior when the TCR recycling point coincides with the APC contact initiation point. (*A*) Domains of the parameter space that determine qualitatively different dynamic scenarios. On the axes, *p_crit_* is the critical TCR density for attachment to the APC, and *k*_ω_ is the rate constant of the contact expansion. Other parameters values are as measured experimentally. *Dark-blue*, no contact develops; *light-blue*, a stable contact develops that is insignificant in size (<30° arc); *yellow*, contact collapses incompletely after exceeding 30° arc transiently; *orange*, contact stabilizes above 30° arc; *red*, infeasible expansion >180° is predicted. *Cross* denotes the parameter combination for the dynamic example shown in panel *B*: *p*_crit_ = 0.09 rad^–1^, *k*_ω_ = 4.7 rad^2^/min. (*B*) Dynamics of the surface TCR distribution *P*(*Φ*, *t*) and contact boundaries *Φ*_1,2_(*t*). TCR density *P* is color-coded in the units of the fraction of the total amount of TCR in the cell per radian of the cell circumference. Solid lines give the position of the contact boundaries *Φ*_1_, *Φ*_2_. Dashed line is the isoline of the critical TCR density for contact expansion, *P* = *p*_crit_. *Φ* is the angular coordinate around the TC, with 270° corresponding to the bottom of the cell where the recycling is assumed to be directed and where the contact with the TCR-binding substrate is initialized in our experiments. *t* is time after the contact initiation (therefore a horizontal line through the plot gives an instantaneous distribution of TCR and the positions of the contact boundaries at the time corresponding to the height at which the imaginary line is drawn).

The model predicted different scenarios, depending on the rate constant of the contact expansion *k*_ω_ and the critical receptor density *p*_crit_ (Fig. 2a). If the critical local density of receptors *p*_crit_ that is required for the expansion of the interface was above the initial density at the site of the initial contact, the contact formation did not commence (the dark-blue region in Fig. 2a). Thus, in this limiting case, the meaning of our parameter *p*_crit_ is exactly the same as in the theory of receptor-mediated cell adhesion [14]. By lowering the critical receptor density our new model could be driven into another regime, wherein the contact expanded to a finite size that was nonetheless insignificant, being less than 30° arc, or 1/12 of the cell circumference (*light-blue* in Fig. 2a). Lowering *p*_crit_ further made possible a significant, albeit transient, expansion (*yellow* in Fig. 2a), and lowering it further still, a contact dynamics that stabilized at a potentially functional contact size >30° arc (*orange* in Fig. 2a). At even lower *p*_crit_, an extreme expansion of the interface over 180° (1/2 of the cell circumference) was predicted. This regime (the red region in Fig. 2a) is infeasible because it would mean engulfment of the TC by the APC, which is not observed in experiments.

The regime of the significant expansion followed by stabilization (*orange* in Fig. 2a) closely resembled the behavior of cells that exhibited the orientation of the RC-MTOC-GA complex to the initial contact point in our experiments [5]. The coupled dynamics of the receptor distribution and the contact area in this regime are plotted in Fig. 2b. The initial contact takes place in the area of the cell surface that is the richest in receptors, because it is where the polarized recycling is directed. The initial expansion is therefore rapid, and the contact zone reaches nearly 150° by 10 min. The expansion then turns into retraction as the receptor density within the contact falls sharply below the critical. Immediately after the commencement of the interface retraction, the zone in which the receptor density is still above critical begins expanding from the center of the interface. This replenishment of the contact with receptors reflects the internalization decrease, which the contact collapse is bringing about by decreasing the area from which the stimulated receptors are internalized at the high ligand-induced rate. By 30 min after the initial contact, the expanding zone of higher-than-critical receptor density meets the slowly collapsing boundary of the contact zone. At that point the receptor density at the boundary equals critical, determining zero instantaneous speed of the boundary. Further collapse of the contact would further decrease the internalization and bring the receptor density above critical, triggering contact expansion. Expansion, however, would increase the zone of the rapid internalization, thus depleting receptors and causing contact collapse. The feedback appears to be rapid enough in the model that only insignificant oscillations of the contact area were observed after 30 min. The contact area was effectively stabilized at about 90° arc, or a quarter of the TC circumference. The TC surface outside the synapse was predicted to be depleted of TCR, while the peak of its surface density is dynamically maintained in the middle of the TC-APC interface through the polarized recycling. Both of these features of the TCR distribution had been observed in experiments [3], [23], [32]–[34].

**Figure 3:**
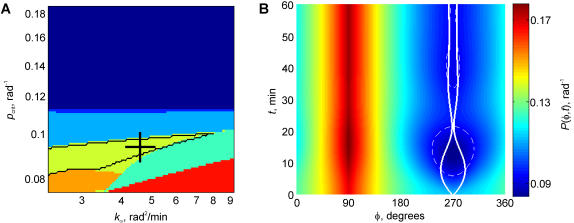
Model behavior when the TCR recycling point is opposite the APC contact initiation point. (*A*) Domains of the parameter space that determine qualitatively different dynamic scenarios. Color coding is the same as in Fig. 2a, with two additional regimes possible in the present case: *medium-blue* (a thin boundary domain separating the light- and dark-blue ones), in which the contact collapses completely without exceeding 30° arc, and *turquoise*, in which the contact collapses completely after exceeding 30° arc transiently. The domain *outlined in black* is determined as an intersection of the domains in this figure and in Fig. 2a: inside the black outline, the formation of a significant (>30°) contact is predicted to be stable if the point of the initial contact coincides with the point of recycling, and transient if the two points are diametrically opposed. Thus, inside the black boundary, the model reproduces our previous experiments. *Cross* denotes the parameter combination for the dynamic example shown in panel *B*, which is the same parameter combination as for the example in Fig. 2b. (*B*) Dynamics of the surface TCR distribution and contact boundaries. The plotting conventions are the same as in Fig. 2b. Recycling is directed to *Φ* = 90° (the top of the cell in the experiments), and the contact is initiated at *Φ* = 270° (cell bottom).

The second experimental situation that we wanted to address was one in which the recycling was directed to the opposite pole of the TC from where the contact was initiated [5]. The simulation in this case was set up identically, except that the recycling was directed to the surface point with the angular coordinate *Φ*_r_ = 90° (top of the cell). This model with the diametrically opposed RC and cell contact exhibited a slightly more diverse set of possible dynamic regimes (Fig. 3a). Most remarkably, in a large domain of the parameter space (turquoise in Fig. 3a), an initial expansion was followed by a complete collapse of the contact area. In another significant parameter-space domain (yellow in Fig. 3a), the initial expansion was followed by a contraction that, although mathematically incomplete, reduced the size of the contact to below 30° arc, or 1/12 of the cell circumference. A contact with a small area is unlikely to be functional because it may not be effective in containing the effector molecules released into the gap between the TC and APC and preventing their diffusion out of the synapse potentially to damage the bystander cells [10]. Most importantly, such a small contact area is unlikely to be detectable in experiments. In our experiments on live cells [5], the refractile cell body would obscure small contacts optically. Thus, in both of these regimes (*turquoise* and *yellow* in Fig. 3a) the model resembled closely the behavior of cells with the RC-MTOC-GA complex diametrically opposed to the contact area, which exhibited the expansion followed by collapse [5]. Also remarkably, the parameter-space domain in which the synapse formation opposite the recycling site was aborted (turquoise and yellow in Fig. 3a) overlapped with the domain in which the synapse formation next to the recycling site was sustained (orange in Fig. 2a). The area of the overlap is outlined in black in Fig. 3a. Within this region of overlap, the model was able to reproduce both experimental observations, using the same parameter values.

As an example, the same combination of parameters as in the dynamic scenario described in detail above, determined only a transient contact formation when the recycling site was diametrically opposed to the contact initiation site. In the latter case, shown in Fig. 3b, the initial expansion of the contact moves its boundaries into the areas on the cell surface which initially had an even higher receptor density. This effect by itself would only speed up the expansion, but it is offset by the intensified internalization of receptors from the contact area. In about 6 min, the receptor density falls below the critical in the middle of the contact. Through the combined action of the position-dependent internalization and lateral diffusion, the depleted zone begins to expand. In 8 min, it overtakes the boundaries of the contact area. At that moment the contact expansion is replaced by collapse. At approximately the same time, one can observe a further rise of the maximum of the PM receptor density next to where the recycling is directed, which reflects the delayed increase of the recycling flux after the internalization was intensified by the contact formation. This density increase is, however, far separated along the PM from the contact area. The contact collapse that is then taking place reduces the internalization flux, slowing down and then reversing the expansion of the local zone depleted of receptors. The contact area collapse, however, is ahead of the delayed collapse of the depleted zone, and the contact area collapse becomes nearly complete by 20 min after the first contact. Shortly after that, the diffusion from the rest of the PM obliterates the area depleted of receptors, so that the receptor density is above critical everywhere again. A secondary expansion of the contact ensues, but this one is very limited and the contact area stabilizes at the insignificant 10° arc through a series of slight further oscillations. We expect that the collapse of the initial wide contact would likely eliminate the cell from the population of attached cells seen in experiments involving chemical fixation accompanied by stirring and replacement of the medium [5], because this degree of collapse should presumably render the contact physically very weak. We also estimate that the limited extent of the secondary contact would preclude its detection in the live-cell studies [5]. The behavior of the theoretical model in this regime can therefore be termed transient contact formation. Thus, the new modeling results demonstrated that the selective contact stabilization conditioned on the polarity of recycling could be explained if the TCR-mediated contact dynamics and their interplay with recycling were taken into account.

### The model predicts migration of the contact area to the recycling pole

**Figure 4:**
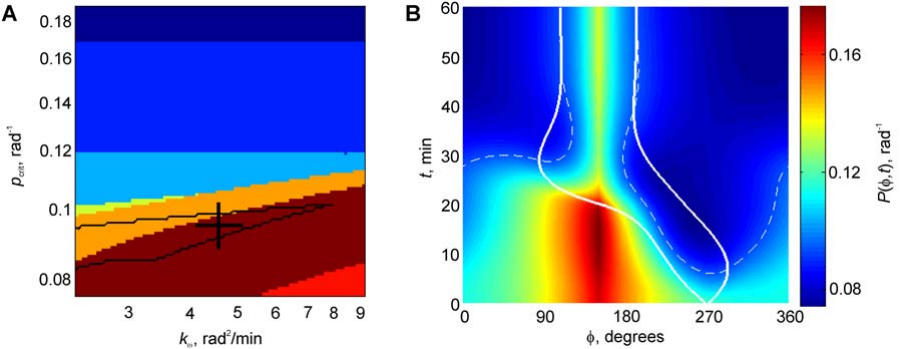
Model behavior when the recycling point is separated from the contact initiation point by 120°. (*A*) Domains of the parameter space that determine qualitatively different dynamic scenarios. Color coding is the same as in Fig. 2a and 3a, with the additional regime denoted *brown*: in this novel regime, the contact area not only stabilizes at >30° arc in size, but also covers the point of recycling no later than in 30 min. The domain *outlined in black* is the same as in Fig. 3a, representing the parameter combinations that predicted model behavior compatible with our previous experiments. Note that the domain predicting the novel behavior (*brown*) overlaps with the domain compatible with the previous experiments (*black outline*). *Cross* denotes the parameter combination for the dynamic example shown in panel *B*, which is the same parameter combination as for the examples in Fig. 2b and 3b. (*B*) Dynamics of the surface TCR distribution and contact boundaries. The plotting conventions are the same as in Fig. 2b and 3b. Recycling is directed to *Φ* = 150°, and the contact is initiated at *Φ* = 270°, which positions are separates by 120° along the TC circumference.

In the general case, the TC coming in contact with the APC may have its RC-MTOC-GA complex located anywhere between the extreme polar positions considered in the previous section. To expand our model analysis to this general case, we assumed that the parameters were the same as in the realistic illustrations of the two structurally extreme cases (Fig. 2b, 3b), and that the recycling point was separated from the initial point of contact by 120° along the model cell circumference (*Φ*_r_ = 150°). In the rest, the modeling procedure in this case was the same as described for the two cases above. However, the dynamic simulation results in the case of the 120° separation of the recycling point from the initial contact were qualitatively different. The model predicted neither stable nor transient symmetric expansion of the contact, but rather lateral migration of the contact to the recycling point, near which it subsequently stabilized. Fig. 4b shows that although the contact expansion in this case is also initially symmetric, the contact boundary expanding away from the recycling point (i.e. to the right in the plot) exhibits rapid deceleration starting at about 4 min, while the boundary moving to the left (to the recycling point) only achieves a nearly constant expansion speed at the same time. The area of the depleted receptors develops in 6 min. In the beginning, it is roughly centered on the point of initial contact. In spite of this, the left-moving contact boundary is able to sweep past the depleted area, while the decelerating right-moving contact boundary crosses into the depleted zone. This switches the right contact boundary from expansion to contraction. Between 10 and 15 min, the contact is predicted to maintain a nearly constant size, while migrating to the left. The speed of migration roughly equals the speed of expansion of the left boundary and the speed of retraction of the right one. The left boundary is thus moving away from the expanding area that is depleted of receptors. It displays a slight acceleration reflecting its advancement into the TCR-rich area next to the recycling point. At about 15 min, the left contact boundary accelerates dramatically, crossing over the recycling point at about 20 min. This is followed by deceleration, as the expanding left boundary is now moving away from the peak of the surface TCR concentration. It eventually crosses into the receptor-depleted area that now envelopes most of the TC surface. From this point on, the overall model dynamics resemble closely the course of the contact stabilization that was seen in the model that started from the contact next to the recycling point (cf. Fig. 2b). Indeed, although the present simulation started from a 120° separation between the recycling point and the contact, the latter migrated by the coordinated expansion to the left and retraction from the right, and covered the recycling zone by 20 min. From that moment the simulation looked essentially as the one that started from the coinciding points of recycling and contact, save for the residual asymmetries and the progressed overall TCR internalization, which only promotes the contact stabilization.

The migration of the contact area over 120° to cover the recycling point was seen in a wide domain of the parameter space, part of which overlaps with the domain that was determined to support the differential stabilization of contact (Fig. 4a). The entire plotted parameter domain that supported the differential stabilization also supported the reorientation of the contact to the recycling point, when they were initially separated by only 60°. At the same time, no part of this domain supported reorientation of the contact to the recycling point, when they were initially separated by as much as 150°. Overall, the model analysis demonstrated that the system spontaneously aligns the contact with the polarity of recycling, thus exhibiting self-stabilizing dynamics even if the recycling pole and the contact area are initially misaligned by up to 120° along the cell circumference.

### Experimental evidence of migration of the contact area to the recycling pole

The predicted migration of the contact area toward the intracellular RC-MTOC-GA complex stipulated that the relative movement of the two structures should be a mutual approach along the cell circumference. The appearance of this relative movement in an experiment would depend on whether the organelle complex or the contact was immobilized in the laboratory coordinates. In our experimental model of the TC-APC interaction, the APC surface is mimicked by the bottom of the observation chamber that is coated with stimulatory antibodies against TCR [5], [9], [20], [21]. In this model system, the contact is immobilized, and therefore the predicted migration of the contact over the surface of the TC was expected to be manifested by a congruous movement of the TC on the immobile substrate in such a way that the eccentric intracellular RC-MTOC-GA complex would become positioned over the cell-substrate contact. The positioning of this organelle complex next to the cell-substrate contact area had been observed in this experimental system as well as in other experimental models of TC-APC interaction [3], [5], [9], [10], [12], [19]. The new theoretical prediction of the migration of the contact to the organelle complex, however, stipulated two more specific features of how this relative position should be achieved. Firstly, as a consequence of the predicted contact migration over the TC surface, the entire TC was expected to be reorienting congruously with respect to the immobilized contact. Secondly, the contact was expected to expand on the immobile substrate asymmetrically, most strongly on the contact side that would be the closest to the RC-MTOC-GA complex. We tested both implications of the model experimentally.

**Figure 5:**
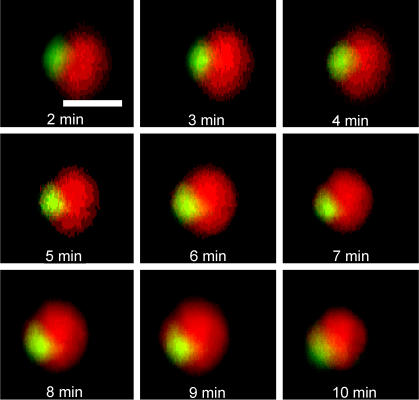
Reorientation of the TC-substrate contact to the GA. The GA is fluorescently labeled green and the nucleus, red. The cell sits on the horizontal, non-fluorescent TCR-binding substrate. A time-sequence of side views of three-dimensional confocal images is shown. The level of the non-fluorescent substrate under the cell is roughly indicated by the 10-µm scale bar in the first image. The GA is seen approaching the substrate on an arc. Since the TCR-binding substrate is immobile in this experimental setup, this kind of cell movement is consistent with migration of the cell-substrate contact area over the cell surface towards the eccentric GA.

The eccentric submembrane pocket of cytoplasm that is occupied by the RC-MTOC-GA complex is complemented to nearly the complete TC volume by the relatively large nucleus. Using three-dimensional time-lapse microscopy, we consistently (57 cells) observed movement of the GA on an arc toward the substrate and the corresponding rotation of the nucleus (Fig. 5). This observation was consistent with the congruous reorientation of the entire TC with respect to the experimentally immobilized contact. It was therefore consistent with the prediction that the contact should migrate over the TC to the RC-MTOC-GA area.

**Figure 6:**
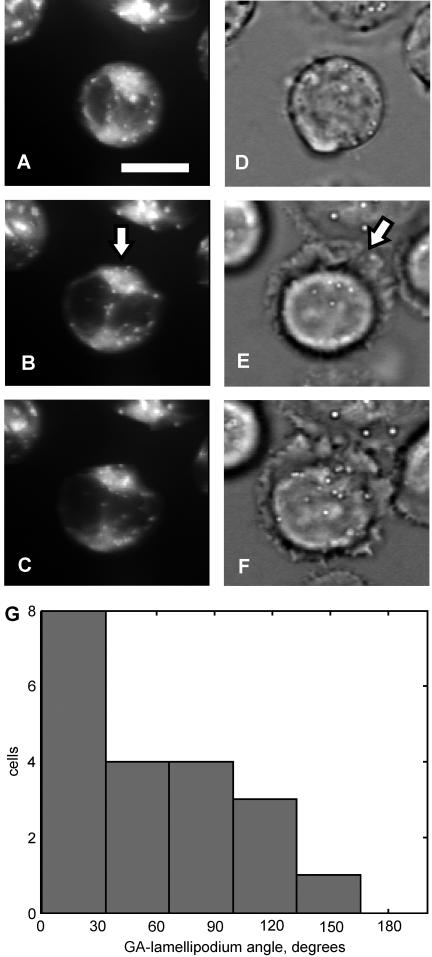
Preferential extension of the TC-substrate contact side that is the closest to the GA. (*A-C*) Time sequence of top views of three-dimensional images of fluorescently labeled GA (*arrow* in *B*) in a TC sitting on the TCR-binding substrate. Scale bar, 10 µm. Time interval, 1 min. (*D-F*) Transmitted-light images of the same cell at the same time points. The median of the asymmetrically extended cell-substrate contact (lamellipodium) is indicated by *arrow* in *E*. Note that the contact extends most strongly on its side that is the closest to the GA, and that its asymmetry can be unequivocally determined at the beginning of extension (*E*). (*G*) Histogram of angles, as seen from the top, between the direction from the cell centroid to the GA centroid and the direction from the cell centroid to the median of the lamellipodium.

The predicted asymmetric extension of the TC interface with the stimulatory substrate could be clearly seen in most cells at the beginning of the contact formation. The extension was most often the strongest on the contact side that was the closest to the GA (Fig. 6a-f). The distribution of the separation between the GA and the median of the initial contact extension supported this observation statistically (Fig. 6g). Thus, the expansion of the contact was biased toward the RC-MTOC-GA complex, consistent with the prediction that the contact should migrate to this submembrane complex over the TC surface.

## Discussion

### Relation of the new model to existing models of TCR recycling

In this paper, we presented a simple, albeit whole-cell-level model of a TC interacting with an APC. The model described the cell-scale TCR distribution, accounted for the structural polarity of the TC, and predicted the position and size of the TC-APC synapse. The model accounts for a number of processes affecting the TCR distribution in the TC: surface diffusion, constitutive as well as ligand-induced internalization, polarized recycling, and dynamic partitioning of the TC surface between the TC-APC interface, where TCR binding takes place, and the rest of the PM. The model used the first-order kinetic formalism for the internalization and recycling that was developed by Geisler et al. [2], [4]. The model development was guided by our previous results obtained with a model that distinguished three fixed compartments between which the surface receptors were considered as partitioned [5]. The previous model had the synapse compartment, and divided the rest of the PM into the imaginary polar cap and equatorial belt. The predictions of that relatively crude model motivated the experiments [5] that the present model was designed to explain. To that end, the surface TCR distribution in the present model was for the first time treated as continuous, and the boundary between the synapse and the rest of the PM, as moving. The new model inherits the feature of the previous model that accounted for the polarity of the TC microtubule cytoskeleton and of the vesicular traffic by considering the receptor recycling as polarized. The polarity is modeled by directing recycling to a special site on the cell surface, which corresponds in the real TC to the PM area adjacent to the polarized submembrane RC-MTOC-GA organelle complex. The new model element – the moving boundary of the synapse – is employed to incorporate the other effects that the structural dynamics of the TC has on the TCR distribution: the absorption of more membrane with the receptors into the synapse region, where the receptors bind ligand and are internalized at the high induced rate. Most importantly, the new model closes the feedback loop between the receptor dynamics and the structural dynamics in the TC by assuming that the speed of the contact boundary is a function of the local receptor density. This assumption is meant to capture the fact that the receptor engagement at the interface stimulates the actin-driven expansion of the interface [19], as well as the more direct impact of the receptor-mediated adhesion [16]. Our assumption of a simple linear relationship between the receptor density and the boundary speed is phenomenological compared to modeling the TC-APC synapse formation on the molecular level [24]. However, by predicting the dynamics of the synapse boundary directly on the cell scale, the present model allows direct comparison with the experiments that were suggested by the previous fixed-compartment model [5].

### Role of binding-induced TCR internalization in TC-APC contact dynamics

The new model results suggest that induced TCR internalization may be a mechanism responsible for limiting and reversing the expansion of the TC-APC interface. Like in the previous models of receptor-mediated adhesion [14], for the initial point contact to begin developing into a significant cell-cell interface area in our model, the local receptor density *P* must be above *p*_crit_. Below this critical value, the local contact expansion rate is considered in our model as negative, which means that the boundary of the TC-APC interface is locally retracting. Importantly, the condition *P*>*p*_crit_ should be met everywhere on the TC surface at the time of its first contact with the APC. Otherwise the contact would only develop where the receptor density in the initial steady state is the highest, which is within the surface area adjacent to the intracellular RC-MTOC-GA organelle complex. The possibility that TC only develop contacts with APC in that special region of the cell surface was initially considered a possible explanation of the observed MTOC polarization to the interface [9]. It was later shown, however, that TC-APC interfaces could form in any orientation to the MTOC [35]. Even though our measurements demonstrated the lower stability of contacts that remained diametrically opposed to the MTOC, their relatively rapid collapse nonetheless followed a period of normal expansion [5]. Thus, the initial expansion of contact from any starting position on the TC surface stipulates that initially, *P* should exceed *p*_crit_ everywhere. One consequence of this requirement is that the expansion will be indefinite, unless the TCR distribution changes with time, dropping below *p*_crit_ somewhere. This consideration underscores the importance of the binding-induced TCR internalization for development of the properly delineated TC-APC interface. For the binding-induced internalization to lower the surface receptor density as required, it must be faster than recycling of the receptors back to the PM, which is indeed the case [2], [4]. Certainly, factors other than the receptor density may also be limiting. Membrane bending is a factor determining the areas of molecular-scale apposition of the TC and APC surfaces [24], and cell deformations should similarly play a role in the development of the cell-scale synapse. The present model does not take into account the development of the internal stress in the TC as it spreads on the TCR-binding surface, which kind of stress was shown to contribute, for example, to limiting the contact area expansion in spreading fibroblasts [36]. Downregulation may also occur downstream of TCR in the signaling cascade to the actin cytoskeleton, whose dynamics contribute to the contact expansion and collapse [19]–[21]. It is nonetheless suggestive of the significant role of TCR internalization that our simple model is able to reproduce the realistically limited interface expansion by assuming only the induced internalization as the limiting mechanism.

### TCR recycling as a mechanism of “proofreading” TC polarization

The fixed-compartment theory [5] explained the accumulation of TCR at the TC-APC interface that had been experimentally observed and linked to recycling [3]. The theory predicted that if recycling was structurally aligned with the cell-cell contact through the commonly observed positioning of the RC-MTOC-GA organelle complex on the synaptic side of the TC [3], [9], [10] , then the surface receptor accumulation in the synapse would be sustained. It also predicted that if the RC-MTOC-GA complex remained diametrically opposed to the synapse in the TC, then the receptor accumulation in the synapse would only be transient. By the nature of the model, these dynamics were predicted for the receptor contents of the synaptic domain that was fixed in size. The predicted receptor dynamics suggested, however, that the TCR-mediated TC-APC interaction could be stabilized if the RC-MTOC-GA complex was polarized to the synapse, and that the synapse could physically collapse if the RC-MTOC-GA complex remained diametrically opposed to it. Since the effector apparatus of TC is structurally a part of the same RC-MTOC-GA organelle complex, an absence of alignment of this intracellular complex with the surface domain of interaction with the target APC should render the TC-APC conjugate non-functional as well as damaging to the bystander cells, at which the immune response would then be structurally directed [10], [13]. We hypothesized that the selective stabilization of only the structurally “correct” cell pairs, as suggested by the selectively sustained TCR accumulation in the fixed synaptic domain of the theory, could be an active “structural proofreading” mechanism for aborting the nonproductive and dangerous TC-APC interactions in case of the structural polarization failure [5]. Such a correcting mechanism would be analogous to the “checkpoint” mechanisms that are sensitive to structural errors in the cell division machinery and abort divisions that would otherwise produce genetically defective daughter cells [22], [37]. The experiments conducted in the model system of Jurkat TC interacting with the artificial TCR-binding substrate demonstrated that the cell-substrate contact was indeed more prone to collapse in cells with the MTOC oriented away from the contact than in cells where it was positioned near the contact [5].

The present model accounting for the dynamic interplay of synapse expansion and receptor dynamics was able to reproduce the differential synapse stability in the experiments closely and in a wide domain of the space of the unknown parameters, while using most parameters as measured in this cell type. This result is nontrivial in view of an alternative dynamics that could be expected based on qualitative intuition. The insufficient initial accumulation, and the insufficient continuous supply of TCR through recycling to the contact site in the “incorrect” orientation should indeed cause a faster onset of contact collapse than in the “correctly” oriented case. With the beginning of the collapse, however, the internalization flux from the contact area should be reduced, and this reduction could act as a feedback mechanism, stabilizing the receptor density and therefore stabilizing the contact. In fact, this is the mechanism whereby the contact is stabilized in the model of the “properly” polarized case as can be seen in Fig. 2. Moreover, a parameter-space domain exists in which both the “properly” and “improperly” polarized cells are predicted to stabilize their synapses: this domain is the intersection of the orange (stabilization) domains in Fig. 2a and 3a, and it resides near the lower-left corner of the plotted parameter space (Fig. 2a, 3a). This model behavior is, however, inconsistent with our previous experimental measurements that demonstrated the dependence of the contact stability on the MTOC orientation. The numerical analysis restricts the behavior consistent with the experiments to the domain outlined in black in Fig. 3a. The nontrivial fact that this parameter-space domain exists demonstrates that the hypothesis of “structural proofreading” in TC-APC interactions is quantitatively consistent, and that the experimentally observed TC dynamics can be understood in a relatively simple spatial-kinetic framework.

### Role of TCR recycling in lateral migration of TC-APC contact

Analyzing numerically the general case of an arbitrary orientation of the recycling polarity with respect to the contact initiation site, we observed an apparent migration of the expanding contact as a whole toward the recycling point on the cell surface. The migration of the contact area around the TC occurred via advancement of the contact boundary that was already closer to the recycling point, while the synapse boundary that was father away from it retracted. After migrating in this manner and finally straddling the recycling point on the PM, the contact was able to stabilize similarly to the contact that was initiated already in this orientation. The contact migration around the cell effectively aligned the intracellular recycling machinery with the contact area as necessary for the stable and productive TC-APC interaction. This behavior exhibited by the model suggested that the role of the receptor recycling may not be restricted to “proofreading” the structural polarity of the TC, but that recycling may also play a more direct role in the genesis of such polarity.

A receptor density gradient created by the constitutive polarized recycling is predicted to already exist on the TC surface when it comes in contact with the APC. Specifically, the model predicts that the boundary of the nascent contact has a higher receptor density on the side of the contact that is more proximal to the RC than on the side of the contact that is more distant from it. Initially, as discussed above, the receptor density on all sides of the contact is above the critical value for contact expansion. To transform the expansion into lateral migration, i.e. into expansion on one side and retraction on the other, the receptor density on the more distant boundary from the RC must be brought down below the critical value. This is achieved in the model via induced internalization within the interface itself: lowering the density at the interface brings the density on the already disadvantaged boundary below the critical value first. At the boundary that is proximal to the RC, the density may remain above critical for the entire duration of the contact migration, as the numerical analysis demonstrates. Two additional factors also come into play. The effect of the recycling proper (return to the PM), although it is delayed because of the long receptor residence time in the RC, is to enhance the gradient for the migration of the interface by transporting more receptors to the area next to the RC, which means closer to the advancing interface boundary. The other effect is due to the migration itself. The advancing boundary moves into the PM region largely unaffected by the induced internalization from within the contact area. This effect is self-accelerating: the faster the advancement of the boundary, the higher the receptor density at the boundary, because the boundary advancement then outpaces the diffusion of the receptors into the depleted region to a higher degree. The retreating boundary at the same time only moves deeper into the region that is depleted of receptors because it has been the interior of the contact for a long time.

### Relation of the contact migration model to models of graded adhesion

Given the role of signaling from TCR to the actin cytoskeleton that powers extension of the TC contact with the TCR-binding surface [19], [20], [38], we are not interpreting the contact formation in our model exclusively as a consequence of receptor-mediated adhesion. The lateral migration of the contact zone is therefore also not exclusively due to the adhesion being stronger where the TCR density is higher. Notwithstanding, the cell-surface binding on one side of the contact area and detachment on the opposite side of it in our model resemble very closely the graded adhesion mechanism in models of cell locomotion. Polarized intracellular trafficking of recycled adhesion receptors was modeled as a mechanism that could generate a gradient of such receptors on the surface of the motile cell, and thus contribute to, drive, and direct the movement of the cell on the substrate to which the receptors have affinity [15]–[18]. The migration is achieved by adhesion on the side of the contact area to which the recycling is directed, and by detachment on the opposite, disadvantaged side. Our model, in comparison, is applied to cells that are not flat but remain on the whole close to spherical in contact with the TCR-binding substrate. More importantly, we do not assume that the polarity of recycling is fixed in the laboratory coordinates. The polarized recycling in this situation is not necessarily directed to a boundary of the contact. It is directed to a point on the surface, whose position relative to the contact zone may change due to the contact dynamics. To contrast our model with the previous models of graded adhesion, consider a situation that is possible only in our model, one in which the point of recycling is initially outside the contact area, albeit near its boundary. Adhesion is then promoted at the nearest contact boundary similarly to the previous models of graded adhesion. However, as the adhesion progresses at the boundary near the recycling point, the incorporation of the cell surface into the cell-substrate contact consumes the free cellular surface, and thus shortens the distance along the cell surface between the contact and the point of receptor recycling. Ultimately this leads in our model to inclusion of the recycling area into the contact area. After the contact boundaries straddle the recycling point, the receptors are distributed to them equally. Both boundaries henceforth have equal propensities for adhesion, and this precludes the recycling point from ever leaving the contact area. On the one hand, this makes our model inapplicable to continuous cell locomotion, which the previous models of graded adhesion addressed [15]–[18]. On the other hand, it constitutes an entirely novel hypothesis for the mechanism of orientation of TCR recycling to the TC-APC contact area, which is observed in experiments [3].

### Relation of the contact migration model to models of MTOC translocation

The effective lateral migration of the TC-APC contact around the TC to the recycling area displayed by the model was the reverse of our starting notion of intracellular migration of the RC, as part of the RC-MTOC-GA complex, to the contact area [3]–[10]. Being a model for the surface receptor distribution and receptor-mediated contact dynamics, our model was not designed to predict the mutual orientation of the contact zone and the intracellular recycling polarity. Rather, a certain orientation of the RC and contact was assumed as part of the initial condition and was implicitly expected to remain constant. That the model nonetheless displayed the spontaneous co-alignment of the receptor-mediated TC-APC contact and the polarized receptor recycling within the TC merits additional discussion.

The driving force of the experimentally observed co-orientation of the RC-MTOC-GA complex with the TC-APC contact is not established. The prominent hypothesis postulates pull between the MTOC and the contact area, generated by cortical molecular motors and mediated by microtubules [39], [40] , whose assembly and disassembly may also be involved in the MTOC repositioning [41], [42]. The pull mechanism would be especially consistent with a relative movement of the MTOC and contact that would be “vectorial”, the MTOC moving to the contact area across the TC interior. Such vectorial translocation was documented using a unique polarization microscope that yielded two-dimensional live images of an experimental system involving TC and APC both partially immobilized on the substrate [39].

In comparison to the pull mechanism, the migration of the contact area around the TC to the region proximal to the RC in our model stipulates, first of all, that the intracellular RC is initially eccentric. The eccentric and essentially submembrane location of the RC-MTOC-GA complex in TC regardless of its orientation to the synapse is documented by numerous data including ours [3], [5], [9], [10]. More specifically, the co-orientation of the RC-MTOC-GA complex with the contact area through migration of the latter over the TC surface stipulates that if the contact is fixed on the TCR-binding surface, congruous rotational movement of the entire TC with respect to the contact should be observed. We document this congruous movement in our experimental system which replaces the APC with the artificial, immobile TCR-binding substrate. Three-dimensional images of TC with the differentially labeled GA and nucleus show that the two organelles, that together comprise most of the TC volume, move as one composite body with respect to the immobilized contact area. This observation can therefore equivalently be described as the contact area moving around the TC as it does in the model.

Any pull mechanism, however, strictly stipulates only the relative movement of the RC-MTOC-GA and the contact, because the two structures are thereby hypothesized to be subject to action and reaction. The vectorial translocation of the RC-MTOC-GA complex through the TC interior is not strictly required; its movement on an arc under the TC surface would also be compatible with its being pulled to the contact area, if, as it appears, the especially massive TC nucleus blocked the way through the interior. Therefore, even though the manner of the relative movement of the contact and the GA that is seen in our time-resolved three-dimensional data is consistent with the migration of the contact to the GA, these data does not argue either way whether it is the contact that is actively moving to the GA or the GA to the contact. This question cannot be answered by observing the relative movement of the two structures.

In search of additional features of the TC dynamics necessary to answer the above question, we paid attention to the shape, rather than the mere position of the TC-substrate contact. The contact was seen expanding asymmetrically, more on the side that was closer to the GA. The asymmetry of the contact expansion suggests that the contact expansion is a driving force of the relative movement of the RC-MTOC-GA complex and the contact. This argument is based on comparing the completeness of the two possible explanations of the experiments. The relative movement of the RC-MTOC-GA complex and the contact can indeed be the same regardless of where the driving force is applied. However, if this force drives the RC-MTOC-GA complex through the cytoplasm, then the asymmetry of the TC-substrate contact expansion remains to be explained. If, on the contrary, the driving force is the contact expansion, then the relative movement and the expansion asymmetry are both explained. We conclude that, to a substantial degree, it should be the TC-APC contact that is moving to the RC-MTOC-GA complex, and not this complex to the contact.

In summary, the presented model generalized previous models of TCR recycling by introducing new level of spatial detail and dynamics to describe the realistic TC-APC interaction. This allowed us to explain quantitatively the previously conducted experiments, formulating the quantitative theory of structural proofreading in TC-APC interactions. Finally, the generalized model predicted a novel mechanism contributing to the overall polarization of TC: the lateral migration of the TC-APC contact area, which aligns the cell-cell contact with the receptor recycling machinery in the TC. The prediction was supported by the new experiments.

## Materials and Methods

### Mathematical modeling

The model is described by the following equations:
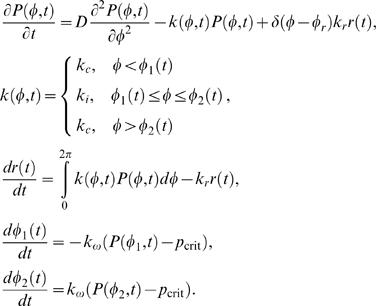
Here, *δ* is the Dirac delta function. The boundary conditions for *P* at *Φ  = *0, 360° are periodic. The initial condition *P*(*Φ*, 0), *r*(0) is the steady-state solution to the modification of the above model, in which the moving boundaries are disregarded and *k* is considered equal to *k*_c_ everywhere. In this way the initial condition for calculating the dynamics after the TC-APC contact represents the steady-state receptor distribution that is achieved in an unstimulated TC before it contacts the APC. The initial condition *Φ*_1,2_(0) = 270° represents initiation of the contact formation on the bottom of the TC, casting our experimental setup (see below) into the conventional polar coordinate system of the model (Fig. 1). In the event that *Φ*_1_(*t*) = *Φ*_2_(*t*) at any *t*>0, the simulation is terminated and its outcome considered contact collapse. In the event that *Φ*_2_(*t*)–*Φ*_1_(*t*)>180°, the simulation is terminated and its outcome considered unfeasibly large contact expansion. The above mathematical formulation is simplified by the fact that in the simulations shown, both *Φ*_1_ and *Φ*_2_ remain between 0 and 360°.

We are using the following values of the rate constants that were measured in Jurkat TC: *k*_c_ = 0.012 min^–1^, *k*_i_ = 0.128 min^–1^, *k*_r_ = 0.055 min^–1^ [2], [4]. Taking the surface TCR diffusion coefficient in Jurkat TC, 0.12 µm^2^/s [11], and the approximate radius 7.5 µm of the Jurkat TCs used in the experiments [5], the angular diffusion constant can be calculated as *D* = (0.12 µm^2^/s)/(7.5 µm/rad)^2^ = 0.128 rad^2^/min. The synapse boundary rate constant *k*_ω_ and the critical receptor density *p*_crit_ required for the local expansion of the synapse are varied in the model analysis. The model was discretized with a uniform Δ*Φ* and solved by the forward Euler method in Matlab software (Mathworks, Natick, MA).

### Experimental procedures

Jurkat cells were grown and prepared for observation essentially as described before [5], [38]. In brief, cells suspended in RPMI1640 growth medium (Invitrogen, Carlsbad, CA) were injected into the observation chamber (LabTek, Brendale, Austria). The chamber bottom was glass pre-coated with anti-TCR antibodies (clone UCHT1, Pharmingen, San Diego, CA). The sedimenting cells were imaged on a Nikon TE 200 inverted microscope (Nikon, Melville, NY) using an ORCA II ERG cooled interline camera (Hamamatsu Photonics, Bridgewater, NJ). The 60x Plan Apochromat water-immersion objective with numerical aperture 1.2 (Nikon) was actuated by a PIFOC 721 piezo-positioner (Physik Instrumente, Auburn, MA). The camera, the objective driver, and a shutter (Vincent Associates, Rochester, NY) were controlled by IPLab software (Scanalytics, Rockville, MD), which was also used for image analysis. The temperature (37°C) was maintained using an ASI 400 air stream incubator (Nevtek, Burnsville, VA). By moving the objective, three-dimensional images were acquired at a formal resolution (voxel size) of 0.22, 0.22, and 0.4 µm in the X, Y, and Z dimensions, Z being along the optical axis and orthogonal to the glass forming the bottom of the observation chamber.

To study movements of the GA and the nucleus, the two organelles were correspondingly labeled with BODIPY FL C5-ceramide and Hoechst 33342 (Molecular Probes, Carlsbad, CA) before the injection into the observation chambers. To that end, after pre-incubation with 5 µM BODIPY FL C5-ceramide-BSA for 10 min, Hoechst 33342 was added to the concentration of 1 µg/ml, and the cells were then incubated with both labels for additional 20 min at 37°C under 5% CO_2_. The images were acquired using a CARV II spinning-disk confocal attachment (BD Biosciences, Franklin Lakes, NJ). At each time point, stacks of images were taken separately in the wavelength channels of the GA and nuclear labels. Each Z-stack was acquired over 7.5 s.

To study mutual orientation of the GA and the initial contact expansion, cells were pre-incubated with the GA fluorescent probe brefeldin-BODIPY558 (Molecular Probes) at 0.1 µM for 20 min. Three-dimensional images were acquired separately on the wavelength corresponding to the fluorescence of the GA label, and in transmitted light showing the area of cell contact with the substrate.

## Supporting Information

Text S1 Russian translation by Ivan Maly.
(1.80 MB DOC)
