# Supplementary material for: A Model for the Interplay of Receptor Recycling and Receptor-Mediated Contact in T Cells
Source: PLoS One. 2007 Jul 25;2(7):e633. doi: 10.1371/journal.pone.0000633 (PMC1920549; doi:10.1371/journal.pone.0000633)
Supplement: Text S1 — Russian translation by Ivan Maly. (1.80 MB DOC) [file pone.0000633.s001.doc]

**Модель взаимодействия рециклирования рецепторов с динамикой опосредованного рецепторами контакта у Т-клеток**

С. Н. Архипов и И. В. Малый

Кафедра вычислительной биологии, медицинский факультет, Университет Питтсбурга, г. Питтсбург, штат Пенсильвания, США

Ключевые слова: *сигнальная система, везикулярный транспорт, микротрубочки, центросома, иммунологический синапс.*

РЕЗЮМЕ

Ориентация органелл внутри Т-клеток (ТК) в направлении антиген-презентирующей клетки (АПК) служит гарантией того, что иммунный ответ будет обладать надлежащей направленностью. Механизмы ориентации, однако, остаются в основном неизученными. Структурная динамика ТК тесно связана с динамикой Т-клеточного рецептора (ТКР), который распознает антиген на поверхности АПК. Связывание ТКР вызывает его интернализацию, за которой следует с задержкой поляризованное рециклирование на плазматическую мембрану через подмембранный рециклирующий компартмент (РК), каковая органелла занимает то же положение в клетке, что и эффекторный аппарат ТК. Связывание ТКР вызывает также расширение зоны контакта между ТК и АПК, которое делает возможным дальнейшее связывание рецепторов. Чтобы проанализировать взаимодействие динамики межклеточного контакта и рецепторов, мы построили новую численную модель. Новая модель воспроизводит наблюдавшиеся в опытах селективную стабилизацию контакта, инициированного вблизи РК, и лишь кратковременное образование контакта, диаметрально противоположного РК. В общем случае, когда контакт между ТК и АПК инициируется в произвольной ориентации к РК, моделирование предсказывает, что динамика контакта и рециклирование рецепторов могут взаимодействовать, приводя фактически к миграции контакта в область на поверхности ТК, соседнюю с подмембранным РК. Посредством микроскопических наблюдений живых клеток в трех измерениях мы получили данные, согласующиеся с таким неожиданным поведением клеток. Мы заключаем, что ТК могут стабилизировать контакт с АПК путем ориентации его по отношению к полярности внутриклеточного транспорта ТКР. Результаты также внушают убеждение, что ориентация органелл ТК, таких как РК и эффекторный аппарат, в направлении АПК может достигаться в отсутствие какого бы то ни было внутриклеточного перемещения самих органелл.

**Источник финансования**: Настоящая работа поддерживалась грантом NIH-GM078332, выданным И.В. Малому.

**Сокращения**: АПК – антиген-презентирующая клетка, АГ – аппарат Гольджи, ЦОМТ – центр организации микротрубочек, ПМ – плазматическая мембрана, РК – рециклирующий компартмент, ТК – Т-клетка, ТКР – Т-клеточный рецептор.

ВВЕДЕНИЕ

Представления о том, что клеточные процессы, как например те, что лежат в основе иммунного ответа, могут включать с трудом поддающиеся интуитивному пониманию взаимодействия между разнородными подпроцессами и компонентами системы и что понимание нами эффектов системного уровня можно значительно улучшить путем применения численного моделирования на компьютере, получают все большее распространение. Модель, представленная в настоящей статье, была разработана для предсказания динамики опосредованных рецепторами межклеточных взаимодействий, исходя из измеренных экспериментально кинетических параметров, и проверки путем сравнения со структурной динамикой клетки, наблюдающейся в эксперименте. На основе предыдущих достижений в области моделирования иммунологической кинетики, введение новых динамических переменных позволило впервые предсказать относительную стабильность и локазизацию иммунологического синапса, которые играют важную роль в иммунологических взаимодействиях клеток. Описанное моделирование направлено главным образом на последовательное и количественное объяснение опытов, которые были проведены ранее, и на постановку новых опытов, результаты которых также приведены в настоящей статье.

Попарные взаимодействия Т-клеток (ТК) с антиген-презентитующими клетками (АПК) иммунной системы и с инфицированными клетками играют центральную роль в клеточном иммунитете. В различных ситуациях, эти взаимодействия могут вызывать различные ответы, включая активацию ТК, индуцирование иммунологической памяти, лизис (разрушение) инфицированных или опухолевых клеток и производство антител [1]. Специфичность ответов определяется на молекулярном уровне специфичностью узнавания антигена, предсталенного на плазматической мембране (ПМ) АПК, Т-клеточным рецептором (ТКР) на ПМ ТК. ТКР непрерывно и активно распределяется в ТК циклом интернализации и реэкспрессии на ПМ [2]. Рециклирование обладает полярностью и вносит вклад в аккумуляцию ТКР в области взаимодействия ТК и АПК [3]. Численные модели рециклирования объясняют распределение ТКР между ПМ и внутриклеточным пулом [4]. Недавно поляризованное накопление ТКР в области взаимодействия ТК и АПК было также описано моделью рециклирования [5]. Однако область взаимодействия в этой модели была фиксированным компартментом. В реальности связывание ТКР в области взаимодействия вызывает расширение самой этой области и, таким образом, вовлечение дополнительного части мембраны с рецепторами во взаимодействие между ТК и АПК [6,7]. В настоящей статье мы описываем пространственно-распределенную кинетическую модель клеточного уровня, которая принимает в расчет взаимодействие между рециклированием ТКР и динамикой опосредованного ТКР межклеточного контакта. Данная модель позволила дать количественное объяснение наших предшествующих опытов и помимо этого продемонстрировала неожиданное поведение, которое мотивировало постановку новых опытов, результаты которых также представлены в настоящей статье.

Предназначение новой модели – отразить ряд черт опосредованного ТКР взаимодейсвия между ТК и АПК в количественных деталях, которые известны из опытов. ТКР конститутивно интернализуется с ПМ. Далее он направляется в составе везикул вдоль микротрубочек в РК [8]. Последний расположен, вместе с аппаратом Гольджи (АГ), возле области схождения микротрубочек, называемой центросомой или центром организации микротрубочек (ЦОМТ). Этот комплекс органелл (РК, ЦОМТ и АГ) обычно лежит в ТК эксцентрично, вблизи ПМ [3,5,9,10]. ТКР рециклируется обратно на ПМ, прилежащую к РК [3], откуда он может диффундировать латерально по всей клеточной поверхности [11]. Когда ТК входит в контакт со специфичной АПК и ТКР на поверхности ТК узнает антиген на поверхности АПК, запускаются два процесса. Во-первых, связывание рецепторов приводит к расширению площади межклеточного контакта (называемого иммунологическим синапсом) и включению в него все большей площади мембраны с рецепторами [6,7]. Во-вторых, интернализация стимулированных рецепторов резко ускоряется [2,4]. Результатом действия этих двух эффектов, которые играют роль, соответственно, положительной и отрицательной обратной связи, может быть либо стабилизация опосредованного ТКР межклеточного контакта, либо его коллапс. Полагают, что значительная продолжительность контакта необходима для того, чтобы взаимодействие клеток было результативным, например, для того, чтобы ТК доставила цитотоксины, убивающие АПК, инфицированную вирусами. Более того, контакт должен быть стабилизирован в области поверхности ТК, которая прилежит к подмембранному комплексу РК, ЦОМТ и АГ, поскольку эффекторный аппарат ТК является частью того же агрегата органелл [9,10,12,13]. Предстваленная здесь численная модель делает попытку предсказать динамику опосредованной ТКР зоны контакта ТК и АПК, исходя из экспериментально измеренных параметров рециклирования ТКР, с целью сравнения предсказания с экспериментально наблюдавшейся динамикой контакта.

Новая модель является континуальным обобщением компартментализированной модели динамики ТКР [5]. В предшествующей модели поверхность ТК, на которой распределены ТКР, рассматривалась не как непрерывная, а как разделенная на три "компартмента", один из которых моделировал зону контакта ТК и АПК. Этот подход позволил описать влияние рециклирования на динамику числа рецепторов в зоне взаимодействия ТК и АПК. Компартментализированная модель не могла, однако, отразить опосредованное рецепторами расширение зоны контакта, которое само по себе изменяет число рецепторов, вовлеченных в межклеточное взаимодействие [7]. Некоторые эффекты расширения зоны контакта были отражены в этой модели как поток рецепторов в контактный компартмент из остальной части ПМ. Измерения показывают, что бòльшая часть такой кажущейся латеральной конвекции ТКР обязана своим происхождением их движению в составе ПМ, становящейся частью расширяющегося межклеточного контакта [7]. Тем не менее, поскольку контактный компартмент в предыдущей численной модели рассматривался как имеющий постоянный размер, феноменологический приток рецепторов в него был предопределен и не зависел от поверхностной плотности рецепторов, уже находящихся в пределах контакта. Вследствие этого, модель не включала положительной обратной связи от связывания рецепторов, которая могла бы противодействовать отрицательной обратной связи, обусловленной интернализацией рецепторов из зоны контакта. В пространственно-непрерывной модели, представленной в настоящей статье, латеральная конвекция ТКР в зону контакта моделируется явно как включение клеточной поверхности в зону контакта по мере расширения последней.

Для моделирования расширения (и сокращения) контакта между ТК и АПК мы вводим движущиеся границы зоны контакта в непрерывную модель поверхности ТК, используя ряд представлений из области моделирования клеточной адгезии, опосредованной рецепторами [14–18]. Адаптируя понятие необходимой для адгезии критической поверхностной плотности рецепторов, взятое из модели прикрепления лейкоцитов [14], мы моделируем границу контакта ТК и АПК как выдвигающуюся, если локальная плотность ТКР выше определенного критического значения, и как отступающую, если она ниже этого значения. В общем случае, скорость границы моделируется линейной функцией локальной поверхностной плотности ТКР. Это простое предположение замыкает петлю положительной обратной связи между плотностью рецепторов в области контакта и вовлечением во взаимодействие новых рецепторов. Оно отражает как тот факт, что связывание ТКР в контактной зоне стимулирует динамику актина, обусловливающую расширение контакта [19–21], так и более прямой вклад опосредованной рецепторами адгезии в формирование контакта [16]. В этом отношении, наша модель является приложением к ТК и ТКР понятия градиентов опосредованной рецепторами клеточной адгезии, создаваемых внутриклеточным транспортом рецепторов [15–18]. Хотя линейная зависимость скорости границы от местной концентрации ТКР является грубым феноменологическим приближением деталей связывания ТКР и активного расширения и сокращения контакта, мы рассматриваем ее как достаточно механистическое предположение в модели, которая имеет целью рассмотрение динамики ТКР на масштабе клетки в ходе взаимодействия ТК с АПК. Принимая в рассмотрение влияние как рециклирования, так и экспансии на потоки ТКР в синапс и из синапса, представленная здесь модель пригодна для анализа динамического взаимодействия этих двух эффектов. Введение в модель новых переменных, задающих положение границ контакта, делает возможным предсказание в явном виде важных в функциональном отношении стабильности и локализации контакта ТК и АПК, исходя из пространственно организованной кинетики рециклирования ТКР. Объяснение опытов с этой точки зрения является основной целью настоящей работы.

Моделирование динамического перераспределения ТКР в синапс и из синапса упрощается возможностью ислючить определенные процессы из рассмотрения в явном виде на основании разделения временных и пространственных масштабов. Времена пребывания рецепторов в ПМ и РК намного короче (минуты) времени жизни рецепторов прежде, чем они деградируются биохимически (часы, [2]). Это позволяет считать полное число рецепторов в клетке постоянным в модели эффектов рециклирования в ходе взаимодействия ТК и АПК на временном масштабе нескольких десятков минут [2]. И наоборот, быстрота внутриклеточного везикулярного транспорта [22] указывает на время транспортировки интернализированного ТКР в РК меньше 1 мин. Это обстоятельство позволяет считать транспорт, как таковой, мгновенным в модели рециклирования ТКР [5]. Было показано, что кривизна мембраны и согласованное распределение с другими трансмембранными молекулами являются важными факторами, влияющими на распреденение ТКР в мелком масштабе в пределах иммунологического синапса [23–25]. Мы опускаем их эффекты в настоящей модели, цель которой – предсказание исключительно крупномасштабного распределения ТКР в целой клетке. Несмотря на то, что исключительное внимание к ТКР и опосредованной этим типом рецепторов динамике клеточного контакта оправдано при моделировании нашей экспериментальной системы, которая включает связывание лишь этого главного типа рецепторов в ТК [19–21], результаты должны быть экстраполированы лишь с осторожностью на реальное взаимодействие ТК с АПК, в которое вовлечены также многие другие типы рецепторов [26]. Наконец, мы делаем упрощающее предположение о комодуляции [2,5], рассматривая все ТКР в пределах синапса как подлежащие интернализации с одной и той же высокой, индуцированной скоростью. В реальности, лишь часть ТКР в синапсе может быть активирована, что было предметом интенсивных теоретических и экспериментальных исследований [27–31]. Наша модель не отражает этой сложной локальной динамики связывания ТКР, сосредотачиваясь вместо этого на распределении ТКР в масштабе клетки.

Нашей первой задачей в настоящей работе было объяснение конкретных опытов [5], проведеных нами ранее на экспериментальной модели, которая заменяет АПК искусственной поверхностью, связывающей ТКР [12,19–21]. С этой целью мы прежду всего воспользовались новым пространственно-кинетическим формализмом для анализа кинетического происхождения стабильности контакта в случае, когда ТК контактирует со связывающей ТКР поверхностью той стороной клетки, рядом с которой находится внутриклеточный РК, и к которой направлено поляризованное рециклирование. Эта экспериментальная ситуация соответствует структурной полярности функциональных и стабильных пар ТК и АПК [3,9,10]. Затем новая модель была применена к экспериментальному случаю, где РК остается диаметрально противоположным клеточной стороне, находящейся в контакте со связывающим ТКР субстратом. Данная экспериментальная ситуация [5] соответствует неудаче структурной поляризации ТК [9,12], которая делает взаимодействие ТК и АПК нефункциональным [10,13]. Мы провели численный анализ, чтобы опрелелить, способна ли модель воспроизвести обращение развития контакта, которое мы наблюдали в этой экспериментальной ситуации, и которое зависело от рециклирования ТКР [5]. Далее, мы обобщили моделирование, рассмотрев начальные условия, в которых РК ориентирован по отношению к месту инициации контакта произвольно. Неожиданные численные результаты в этом случае заставили нас пересмотреть некоторые предположения, касающиеся причинно-следственных отношений в поляризации ТК, и провести новые опыты, которые подтвердили предсказания модели.

РЕЗУЛЬТАТЫ

**Модель воспроизводит различную стабильность контакта в зависимости от полярности рециклирования**

Пространственная организация модели показана диаграмматически на рис. 1 (см. тж. полную математическую формулировку в разделе «Материалы и методы»). Модель описывает перераспределение ТКР в ТК, взаимодействующей с АПК, и динамику контакта между ТК и АПК. С этой целью, поверхность ТК моделируется окружностью, на которой распределен ТКР, и часть которой обозначает площадь контакта с АПК. Положение на поверхности задается угловой координатой *φ*. Она отсчитывается между 0 и 360° как принято в полярной системе координат, так что верх клетки имеет координату *φ* = 90° в то время как низ, где всегда инициируется контакт со связывающей ТКР поверхностью в нашей экспериментальной системе, имеет координату *φ* = 270°. Две границы, которые отделяют дугу, обозначающую зону контакта ТК с АПК (или с биомиметической связывающей ТКР поверхностью), могут перемещаться. Их мгновенные положения на окружности задаются угловыми координатами *φ*_1_ и *φ*_2_. Распределение ТКР по поверхности описывается математически функцией плотности *P*(*φ*), которая обозначает локальную поверхностную плотность (концентрацию) рецепторов в положении с координатой *φ*. Зависяшая от времени переменная *r* описывает количество ТКР во внутриклеточном РК. Поток со всей ПМ (интернализация) направлен в РК, в то время как поток из РК (собственно рециклирование) направлен в единственную, фиксированную точку на ПМ, положение которой задается *φ_r_*. Эта точка является идеализацией области клеточной поверхности, которая прилегает к РК и к которой вследствие этого направлено рециклирование. Константа скорости рециклирования обозначена *k*_r_. ТКР на поверхности претерпевает латеральную диффузию с коэффициентом диффузии *D*. Важной чертой модели является сопряжение динамики ТКР с динамикой контакта. Между двуми границами области контакта, интернализация рецепторов протекает с высокой, индуцированной лигандом константой скорости *k*_i_, в то время как в остальной модельной ПМ она протекает с относительно низкой, конститутивной константой скорости *k*_c_. Принципиально новой чертой модели является то, что эти две границы двигаются латерально по поверхности клетки со скоростями, зависящими от местной плотности ТКР. Мы прибегли к упрощающему предположению, что мгновенная скорость границы является линейной функцией местной плотности рецепторов. Опосредованное рецепторами образование контакта между ТК и АПК таким образом описывается двумя варьируемыми параметрами: критической плотностью рецепторов *p*_крит_ и константой угловой скорости *k*_ω_. Предполагается, что если локальная плотность рецепторов на границе контакта выше *p*_крит_, то граница продвигается вперед, расширяя зону контакта. Если она ниже *p*_крит_, то граница отступает, делая зону контакта более узкой. Насколько быстро граница продвигается или отступает, в зависимости от отклонения местной плотности рецепторов от *p*_крит_ – определяется константой скорости *k*_ω_. Вычислительные детали модели описаны в разделе «Материалы и методы».

В первом модельном случае, мы предположили, что комплекс РК, ЦОМТ и АГ поляризован к области контакта с самого начала. Эта ситуация моделировалась экспериментально и стабильность контакта в ней была измерена [5]. В условиях опыта, контакт инициировался на «дне» приблизительно шарообразной ТК. Для моделирования полярности рециклирования в данном случае, мы задали положение поверхностной точки рециклирования внизу клетки (*φ*_r_ = 270°).

Распределение ТКР в момент инициации контакта (*t* = 0) должно представлять собой стационарное распределение ТКР в изолированной ТК. Такое распределение может быть получено как стационарное решение варианта модели без границ контакта и с интернализацией, которая протекает с низкой, конститутивной скоростью повсюду на клеточной поверхности. В таком базальном стационарном состоянии, ТКР предсказывается распределенным в соотношении 82:18 между поверхностью и внутриклеточным пулом. Это соотношение очень хорошо согласуется с предшествующей моделью, не принимавшей поверхностное распределение во внимание [4]. Вследствие поляризованного рециклирования, предсказываемое настоящей моделью распределение поверхностного ТКР в базальном стационарном состоянии неравномерно. Предсказанная повехностная плотность ТКР в 1,5 раза выше внизу клетки, в области, куда направлено рециклирование, чем на противоложном, верхнем полюсе клетки. Такая степень поляризации поверхностных ТКР близко согласуется с предшествующей моделью, которая не была пространственно непрерывной, но различала три воображаемых области на ПМ [5].

Для того, чтобы начать моделирование формирования опосредованного ТКР контакта, мы ввели две границы зарождающегося контакта внизу клетки (*φ*_1_,_2_(0) = 270°). С этого момента, границы двигались в соответствии с плотностью рецепторов в месте их положения, и интернализация рецепторов между границами протекала с высокой, индуцированной лигандом скоростью.

Модель предсказывала различные сценарии в зависимости от константы скорости экспансии контакта *k*_ω_ и критической плотности рецепторов *p*_крит_ (рис. 2а). Если критическая локальная плотность рецепторов *p*_крит_, требуемая для экспансии области взаимодействия была выше первоначальной плотности в месте инициации контакта, формирование контакта не могло начаться (темно-синяя область на рис. 2а). Таким образом, в этом лимитирующем случае, физический смысл параметра *p*_крит_ в точности тот же, что и в теории клеточной адгезии, опосредованной рецепторами. Снижением критической плотности рецепторов наша новая модель могла быть переведена в другой режим, в котором контакт расширялся до конечного размера, который был тем не менее незначительным, меньше, чем 30° дуги, или 1/12 клеточной окружности (*светло-синий* на рис. 2а). Дальнейшее снижение *p*_крит_ делало возможным значительную, хотя и преходящую, экспансию (*желтый* на рис. 2а), и еще более глубокое снижение – динамику контакта, которая стабилизировалась на потенциально функциональном размере контакта, >30° дуги (*оранжевый* на рис. 2а). При еще более низкой *p*_крит_ было предсказано экстремальное расширение зоны контакта, превышающее 180° (1/2 окружности клетки). Последний режим (красная область на рис. 2а) является неправдоподобным, поскольку он означал бы поглощение ТК внутрь АПК, чего не наблюдается в опытах.

Режим значительного расширения, за которым следовала стабилизация (*оранжевый* на рис. 2а), имел большое сходство с поведением клеток, в которых комплекс РК, ЦОМТ и АГ был ориентирован к точке первоначального контакта в наших опытах [5]. Сопряженная динамика распределения рецепторов и площади контакта в этом режиме показана на рис. 2б. Первоначальный контакт имеет место в области клеточной поверхности, которая наиболее богата рецепторами, поскольку туда направлено поляризованное рециклирование. Начальная экспансия поэтому протекает быстро и ширина контакта достигает 150° к 10 мин. Расширение затем сменяется сужением, когда плотность рецепторов в пределах контакта резко падает ниже критической. Непосредственно после начала сужения контакта зона, в которой плотность рецепторов все еще превышает критическую, начинает расширяться от центра контакта. Это пополнение контакта рецепторами отражает уменьшение интернализации, которое вызывается коллапсом контакта, уменьшающим площадь, из которой стимулированные рецепторы интернализуются с высокой, индуцированной лигандом скоростью. К 30 мин после первоначального соприкосновения, расширяющаяся зона плотности рецепторов, превышающей критическую, встречается с медленно коллапсирующей границей области контакта. В этот момент плотность рецепторов на границе контакта равняется критической, что обусловливает нулевую мгновенную скорость границы. Дальнейший сокращение зоны контакта понижало бы интернализацию и приводило бы к росту плотности рецепторов выше критической, вызывая таким образом экспансию контакта. Его экспансия, однако, увеличивала бы зону быстрой интернализации, таким образом обедняя контакт рецепторами и вызывая его коллапс. Обратная связь представляется достаточно быстрой в данной модели, так что лишь незначительные осцилляции площади контакта наблюдаются после 30 мин. Зона контакта фактически стабилизируется на приблизительно 90° дуги, или четверти окружности ТК. Модель предсказывает, что поверхность ТК за пределами синапса обеднена рецепторами, в то время как пик их поверхностной плотности динамически поддерживается в середине области взаимодействия с АПК поляризованным рециклированием. Обе черты распределения ТКР наблюдались в опытах [3,23,32–34].

Вторая экспериментальная ситуация, которую мы хотели проанализировать, была той, в которой рециклирование направлено к противоположному полюсу по отношению к месту инициации контакта [5]. Симуляция в этом случае была проведена таким же образом, за исключением того обстоятельства, что рециклирование было направлено в точку поверхности с угловой координатой *φ*_r_ = 90° (верх клетки). Такая модель с диаметрально противоположными РК и клеточным контактом продемонстрировала несколько более разнообразный набор возможных динамических режимов (рис. 3а). Особенно примечательно было то, что в широкой области пространства параметров (бирюзовой на рис. 3а), за первоначальным расширением следовал полный коллапс зоны контакта. В другой значительной области пространства параметров (желтой на рис. 3а), первоначальное расширение сменялось сокращением, которое, хотя оно оставалось математически неполным, сводило размер контакта к значениям ниже 30° дуги, или 1/12 окружности клетки. Контакт такого размера вряд ли был бы функциональным, поскольку он не был бы в состоянии задерживать эффекторные молекулы, высвобождаемые в зазор между ТК и АПК, и предотвращать их диффузию за пределы синапса достаточно эффективно, что потенциально могло бы приводить к повреждению невовлеченных в иммунологическое взаимодействие окружающих клеток [10]. Важно и то, что контакт столь малого размера вряд ли был бы обнаружен в экспериментах. В наших опытах на живых клетках [5] обнаружение контактов малого размера затруднено светопреломлением в теле клетки. Таким образом, в обоих этих режимах (*бирюзовый* и *желтый* на рис. 3а) модель близко напоминала поведение клеток с комплексом РК, ЦОМТ и АГ, диаметрально противоположным зоне контакта, которые демонстрировали экспансию, сменявшуюся коллапсом [5]. Также примечательно, что область в пространстве параметров, в которой формирование синапса, противолежащего месту рециклирования, прерывается (*бирюзовый* и *желтый* на рис. 3а) перекрывается с областью, в которой формирование синапса возле места рециклирования является устойчивым (оранжевой на рис. 2а). Область перекрывания очерчена черным на рис. 3а. В пределах этой области перекрывания, модель в состоянии воспроизвести оба экспериментальных наблюдения, используя одни и те же значения параметров.

В качестве примера, та же комбинация параметров, что и в динамическом сценарии, детально описанном выше, задает лишь временное формирование контакта, если место рециклирования диаметрально противоположно месту инициации контакта. В последнем случае, показанном на рис. 3б, первоначальное расширение контакта приводит к продвижению его границ в область клеточной поверхности, которая исходно имеет еще более высокую плотность рецепторов. Данный эффект сам по себе только ускорил бы экспансию, но он компенсируется интенсифицированной интернализацией рецепторов из зоны контакта. Приблизительно через 6 мин, плотность рецепторов падает в середине контакта ниже критической. Путем совместного действия зависящей от положения интернализации и латеральной диффузии, обедненная область начинает расширяться. Через 8 мин, она обгоняет границы зоны контакта. В этот момент экспансия контакта сменяется коллапсом. Приблизительно в то же время можно наблюдать дальнейший рост максимума плотности рецепторов на ПМ возле того места, куда направлено рециклирование, что отражает возрастание потока рециклирования, которое следует с задержкой за интенсификацией интернализации формированием контакта. Этот рост плотности, однако, далеко отстоит вдоль ПМ от зоны контакта. Происходящий в это время коллапс контакта снижает интернализационный поток, замедляя и затем обращая расширение местной зоны, обедненной рецепторами. Коллапс зоны контакта, однако, опережает задержанный коллапс обедненной зоны, так что коллапс зоны контакта становится практически полным к 20 мин после первого соприкосновения. Вскоре после этого, диффузия с остальной части ПМ стирает зону, обедненную рецепторами, так что плотность рецепторов снова становится повсюду выше критической. Начинается вторичная экспансия контакта, но она весьма ограничена и зона соприкосновения стабилизируется на незначительных 10° дуги через серию слабых дальнейших осцилляций. Мы ожидаем, что коллапс первоначального широкого контакта должен был бы исключить клетку из популяции конъюгированных клеток в опытах, включающих химическую фиксацию, сопровождающуюся перемешиванием и заменой среды [5], поскольку такая степень коллапса должна по идее сделать контакт физически весьма слабым. Мы также оцениваем ограниченную степень вторичной экспансии как такую, какая сделала бы невозможным ее обнаружение в исследованиях на живых клетках [5]. Поведение теоретической модели в настоящем режиме может быть поэтому названо кратковременным установлением контакта. Таким образом, новые результаты моделирования показывают, что селективная стабилизация контакта в зависимости от полярности рециклирования может быть объяснена, если включить в расмотрение динамику опосредованного ТКР контакта и ее взаимодействие с рециклированием.

**Модель предсказывает миграцию зоны контакта к полюсу рециклирования**

В общем случае, комплекс РК, ЦОМТ и ГА может располагаться в ТК, приходящей в соприкосновение с АПК, где бы то ни было между крайними, полярными положениями, рассмотренными в предыдущем разделе. Чтобы расширить анализ нашей модели и рассмотреть этот общий случай, мы предположили, что параметры оставались теми же самыми, что и в реалистичных иллюстрациях двух структурно крайних случаев (рис. 2б, 3б), и что точка рециклирования была отделена от первоначальной точки контакта 120° вдоль окружности модельной клетки (*φ*_r_ = 150°). В остальном, процедура моделирования в этом случае была той же, что была описана для двух случаев выше. Однако результаты динамической симуляции в случае 120-градусного разделения точки рециклирования и первоначального контакта оказались качественно иными. Модель не предсказала ни стабильного, ни кратковременного симметричного расширения контакта. Вместо этого, была предсказана латеральная миграция контакта к точке рециклирования, возле которой он затем стабилизировался. Рис. 4б показывает, что несмотря на то, что расширение контакта в настоящем случае вначале также симметрично, граница контакта, которая в своем расширении удаляется от точки рециклирования (т.е., движется вправо на графике), проявляет быстрое замедление, начиная с 4 мин, в то время как граница, перемещающаяся влево (в направлении точки рециклирования), лишь достигает приблизительно постоянной скорости расширения. Зона, обедненная рецепторами, развивается через 6 мин. Вначале, она приблизительно центрирована на точке первого соприкосновения. Несмотря на это, граница контакта, движущаяся влево, избегает обедненной области, в то время как замедляющаяся граница, движущаяся вправо, входит в обедненную область. Это приводит к смене продвижения правой границы ее отступлением. Между 10 и 15 мин, модель предсказывает сохранение контактом почти постоянного размера в то же самое время, как он мигрирует влево. Скорость миграции приблизительно равна скорости экспансии левой границы и отступления правой. Левая граница таким образом движется в направлении от расширяющейся области, бедной рецепторами. Она демонстрирует постепенное ускорение, отражающее ее продвижение в область, богатую ТКР вблизи точки рециклирования. Приблизительно через 15 мин, левая граница резко ускоряется и пересекает точку рециклирования через 20 мин. За этим следует замедление по мере того, как расширяющаяся левая граница удаляется от пика поверхностной концентрации ТКР. Наконец, она входит в область бедной рецепторами области, которая к тому времени обволакивает большую часть поверхности ТК. С этого времени, динамика в целом близко напоминает ход стабилизации контакта, наблюдавшейся в модели, которая исходила из контакта возле точки рециклирования (ср. рис. 2б). В самом деле, несмотря на то, что настоящая симуляция начиналась с 120-градусного разделения точки рециклирования и контакта, последний мигрировал посредством координированной экспансии слева и ретракции справа и накрыл зону рециклирования к 20 мин. С этого момента симуляция выглядела в основных чертах так же, как и симуляция, начинавшаяся с совпадающих точек рециклирования и контакта, если не придавать значения остаточной асимметрии и в целом более продвинутой интернализации ТКР, которая только способствует стабилизации контакта.

Миграция зоны контакта на 120°, чтобы накрыть точку рециклирования, наблюдалась в широкой области пространства параметров, часть которой перекрывается с областью, которая поддерживала селективную стабилизацию контакта (рис. 4а). Вся область, показанная на графике, которая поддерживала селективную стабилизацию, поддерживала также переориентацию контакта к точке рециклирования, если они были исходно разделены лишь 60°. В то же время, никакая часть этой области не поддерживала переориентацию контакта к точке рециклирования, если они были исходно разделены по меньшей мере 150°. В целом, анализ модели продемонстрировал, что система самопроизвольно совмещает контакт с полюсом рециклирования, проявляя таким образом самостабилизирующую динамику, даже если полюс рециклирования и зона контакта исходно разориентированы на 120° вдоль окружности клетки.

**Экспериментальное свидетельство миграции зоны контакта к полюсу рециклирования**

Предсказанная миграция зоны контакта к внутриклеточному комплексу РК, ЦОМТ и АГ подразумевает, что относительное движение этих двух структур должно быть взаимным сближением вдоль клеточной окружности. Вид их относительного движение в опыте будет зависеть от того, какая из них – комплекс органелл или контакт – иммобилизирована в лабораторной системе координат. В нашей экспериментальной модели взаимодействия ТК и АПК поверхность АПК моделируется дном камеры для наблюдений, которое покрыто стимулирующими антителами к ТКР [5,9,20,21]. В этой модельной системе, контакт иммобилизирован, и поэтому предсказанная миграция контакта по поверхности ТК должна была бы проявляться как согласованное движение ТК на неподвижном субстрате таким образом, что эксцентричный внутриклеточный комплекс РК, ЦОМТ и АГ становился бы расположенным над контактом между клеткой и субстратом. Расположение этого комплекса органелл около зоны контакта клетки с субстратом наблюдалось в этой экспериментальной системе, так же, как и в других экспериментальных моделях взаимодействия ТК и АПК [3,5,9,10,12,19]. Новое теоретическое предсказание миграции контакта к комплексу органелл, однако, подразумевает две более специфические черты того, как это относительное положение должно достигаться. Во-первых, как следствие предсказанной миграции контакта по клеточной поверхности, вся ТК должна переориентироваться согласованно по отношению к иммобилизованному контакту. Во-вторых, контакт должен вытягиваться на неподвижном субстрате асимметрично: наиболее сильно на стороне контакта, которая наиболее близко расположена к комплексу РК, ЦОМТ и АГ. Мы проверили оба предсказания экспериментально.

Эксцентрично расположенный подмембранный карман цитоплазмы, который занят комплексом РК, ЦОМТ и АГ, дополнен до почти полного объема ТК сравнительно большим ядром. С использованием трехмерной цейтраферной микроскопии, мы обычно наблюдали (в 57 клетках) движение АГ по дуге в направлении субстрата и соответствующее вращение ядра (рис. 5). Это наблюдение согласуется с конгруэнтной переориентацией всей ТК по отношению к экспериментально иммобилизированному контакту. Поэтому оно совместимо с предсказанием, что контакт должен мигрировать вокруг ТК к области РК, ЦОМТ и АГ.

Предсказанное асимметричное вытягивание области взаимодействия ТК со стимулирующим субстратом было легко заметно в большинстве клеток в начале формирования контакта. Вытягивание было чаще всего наиболее сильным на той стороне контакта, которая прилегала наиболее близко к АГ (рис. 6а-е). Распределение углового расстояния между АГ и медианой первоначального выпячивания контакта подтверждало это наблюдение статистически (рис. 6ж). Таким образом, расширение контакта отклонялось в направлении комплекса РК, ЦОМТ и АГ, в согласии с предсказанием о том, что контакт должен мигрировать к этому подмембранному комплексу по поверхности ТК.

ОБСУЖДЕНИЕ

**Отношение новой модели к существующим моделям рециклирования ТКР**

В настоящей статье мы сформулировали простую, но тем не менее клеточного уровня модель ТК, взаимодействующей с АПК. Модель описывает распределение ТКР в масштабе клетки, отражает структурную полярность ТК и предсказывает положение и размер синапса между ТК и АПК. Модель отражает многие процессы, оказывающие влияние на распределение ТКР в ТК: поверхностную диффузию, конститутивную и индуцированную лигандом интернализацию, поляризованное рециклирование и динамическое перераспределение поверхности ТК между областью взаимодействия с АПК, где имеет место связывание ТКР, и остальной ПМ. Модель использует кинетический формализм первого порядка для интернализации и рециклирования, который был разработан Гейслером и др. [2,4]. Разработка модели руководствовалась результатами, полученными нами ранее на модели, которая различала три фиксированных компартмента и рассматривала перераспределение между ними поверхностных рецепторов [5]. Предшествующая модель имела синаптический компартмент и разделяла остальную часть ПМ на условные полярную шапку и экваториальный пояс. Предсказания этой сравнительно грубой модели мотивировали постановку опытов [5], которые представленная в настоящей статье модель была призвана объяснить. С этой целью, поверхностное распределение ТКР в настоящей модели впервые рассматривалось как непрерывное и граница между синапсом и остальной ПМ – как подвижная. Новая модель унаследовала черту предыдущей модели, которая отражала полярность микротрубочкового цитоскелета ТК и везикулярного транспорта, рассматривая рециклирование рецепторов как поляризованное. Полярность моделируется путем направления рециклирования в особое место на клеточной поверхности, которое соответствует в реальной ТК области ПМ, прилежащей к поляризованному подмембранному комплексу РК, ЦОМТ и АГ. Новый элемент модели – движущаяся граница синапса – используется для учета другого эффекта, оказываемого структурной динамикой ТК на распределение ТКР: включения дополнительного количества мембраны с рецепторами в состав синапса, где рецепторы связывают лиганд и интернализируются с высокой скоростью. Наиболее существенно при этом то, что новая модель замыкает петлю обратной связи между динамикой рецепторов и структурной динамикой в ТК, предполагая, что скорость границы контакта является функцией локальной плотности рецепторов. Это допущение имеет целью отразить тот факт, что связывание рецепторов в области клеточного взаимодействия стимулирует расширение области взаимодействия путем модуляции актинового цитоскелета [19], а также более прямой вклад опосредованной рецепторами адгезии [16]. Наше предположение о простой линейной зависимости между плотностью рецепторов и скоростью границы является феноменологическим сравнительно с моделированием формирования синапса между ТК и АПК на молекулярном уровне [24]. Тем не менее, предсказывая динамику границы синапса непосредственно на масштабе клетки, представленная здесь модель делает возможным прямое сравнение с опытами, которые были подсказаны предшествующей моделью с фиксированными компартментами [5].

**Роль индуцированной связыванием интернализации в динамике контакта ТК и АПК**

Полученные на новой модели результаты наводят на мысль, что индуцированная интернализация ТКР может быть механизмом, отвечающим за ограничение и обращение расширения области взаимодействия между ТК и АПК. Как и в предшествующей модели опосредованной рецепторами адгезии [14], для того, чтобы первоначальный точечный контакт начал развиваться в значительную площадь межклеточного взаимодействия, локальная плотность рецепторов на нашей модели должна превышать *p*_крит_. Ниже этого критического значения, локальная скорость расширения контакта рассматривается в нашей модели как отрицательная, что означает, что граница области взаимодействия ТК и АПК отступает в этом месте. Представляется важным тот факт, что условие *P* > *p*_крит_ должно удовлетворяться повсюду на поверхности ТК во время ее первого соприкосновения с АПК. Иначе контакт развивался бы только там, где плотность рецепторов в исходном стационарном состоянии наиболее высока, то есть в пределах области поверхности, прилежащей к внутриклеточному комплексу органелл РК, ЦОМТ и АГ. Возможность того, что ТК развивают взаимодействие с АПК лишь в этом особом районе клеточной поверхности изначально рассматривалась в качестве возможного объяснения наблюдаемой поляризации ЦОМТ к зоне взаимодействия [9]. В дальнейшем, однако, было показано, что взаимодействие ТК и АПК может развиваться в любой ориентации к ЦОМТ [35]. Несмотря на то, что наши измерения продемонстрировали более низкую стабильность контактов, которые оставались диаметрально противоположными ЦОМТ, их относительно быстрый коллапс тем не менее следовал за периодом нормального расширения [5]. Таким образом, первоначальное расширение контакта из любого исходного положения на поверхности ТК требует, чтобы вначале *Р* была повсюду выше *p*_крит_. Одним из следствий этого требования является то, что расширение будет неограничено, если только распределение ТКР не изменяется со временем, падая где бы то ни было ниже *p*_крит_. Это соображение подчеркивает важность индуцированной связыванием интернализации для развития надлежащим образом ограниченной площади взаимодействия ТК и АПК. Для того, чтобы индуцированная связыванием интернализация понизила поверхностную плотность рецепторов требуемым образом, она должна протекать с более высокой скоростью, чем рециклирование рецепторов назад на ПМ, что в самом деле имеет место [2,4]. Разумеется, иные факторы нежели плотность рецепторов могут быть лимитирующими. Изгиб мембраны является одним из факторов, определяющих зоны аппозиции поверхностей ТК и АПК на молекулярном масштабе [24], и клеточные деформации должны подобным же образом играть роль в развитии синапса на масштабах клетки. Настоящая модель не принимает во внимание развитие внутренних напряжений в ТК по мере того, как она распластывается на связывающей ТКР поверхности, какого рода напряжения, как было показано, вносят вклад в ограничение расширения площади контакта у распластывающихся фибробластов [36]. Затухание сигнала может также иметь место ниже ТКР в сигнальном каскаде, ведущем к актиновому цитоскелету, динамика которого вносит вклад в экспансию и коллапс контакта [19–21]. Тем не менее то, что наша простая модель в состоянии воспроизвести реалистично ограниченное расширение области взаимодействия, предполагая лишь индуцированную интернализацию как лимитирующий механизм, наводит на размышления, что роль интернализации ТКР в этом процессе может быть значительной.

**Рециклирование ТКР как механизм «проверочного считывания» поляризации ТК**

Теория с фиксированными компартментами [5] объяснила накопление ТКР в области контакта ТК и АПК, которое наблюдалось и было связано с рециклированием в опытах [3]. Теория предсказала, что если рециклирование структурно направлено к межклеточныму контакту посредством обычно наблюдаемого расположения комплекса органелл РК, ЦОМТ и АГ на синаптической стороне ТК [3,9,10], то накопление поверхностных рецепторов в синапсе должно быть устойчивым. Она также предсказала, что если комплекс РК, ЦОМТ и АГ остается диаметрально противоположным синапсу в ТК, то накопление рецепторов в синапсе должно быть лишь временным. В силу характера модели, такого рода динамика была предсказана для содержания рецепторов в синаптической области, имевшей фиксированный размер. Предсказанная динамика рецепторов тем не менее указывала, что опосредованное ТКР взаимодействие ТК с АПК могло быть стабилизировано, если комплекс РК, ЦОМТ и АГ был поляризован к синапсу, и что синапс мог физически коллапсировать, если комплекс РК, ЦОМТ и АГ оставался ему диаметрально противоположен. Ввиду того, что эффекторный аппарат ТК структурно входит в состав того же комплекса органелл, отсутствие сонаправленности этого внутриклеточного комплекса с поверхностной областью взаимодействия с АПК-мишенью должна делать конъюгат ТК и АПК нефункциональным, а также наносящим ущерб невовлеченным во взаимодействие окружающим клеткам, на которые в этом случае будет стурктурно направлен иммунный ответ [10,13]. Мы выдвинули гипотезу, согласно которой стабилизация лишь структурно «правильных» пар клеток, на возможность которой указывала селективность устойчивого накопления ТКР в фиксированном синаптическом домене теории, могла служить в качестве активного механизма «проверочного считывания» для аварийного прекращения непродуктивных и опасных взаимодействий ТК с АПК в случаях неудачи структурной поляризации [5]. Такого рода корректирующий механизм был бы аналогичен механизмам типа «пропускного пункта», чувствительным к структурным неточностям в аппарате клеточного деления и прекращающим деления, которые иначе приводили бы к образованию генетически неполноценных дочерних клеток [22,37]. Опыты, проведенные на модельной системе ТК линии Юркат, взаимодействующих с искусственным, связывающим ТКР субстратом, продемонстрировали, что контакт клетки с субстратом в самом деле проявлял большую склонность к коллапсу в клетках, где ЦОМТ был ориентирован в противоположную сторону от контакта, чем в клетках, где он располагался около контакта [5].

Представленная здесь модель, принимающая в расчет динамическое взаимодействие расширения контакта и динамики рецепторов, в состоянии воспроизвести известную из опытов дифференциальную стабильность синапса с достаточной точностью и в широком диапазоне неизвестных параметров, в то время как значения большинства параметров были такие, как были измерены в данном типе клеток. Этот результат представляется нетривиальным, если рассмотреть альтернативную динамику, которую можно было бы ожидать, опираясь на качественные, интуитивные рассуждения. Недостаточная начальная аккумуляция и недостаточный продолжающийся приток ТКР посредством рециклирования в зону контакта в «неправильном» положении в самом деле должна вызвать более скорое начало коллапса контакта сравнительно со случаем «правильной» ориентации. С началом коллапса, однако, интернализационный поток из области контакта должен уменьшиться, и это уменьшение может сыграть роль механизма обратной связи, стабилизируя плотность рецепторов и, таким образом, стабилизируя контакт. На самом деле, именно таков механизм, посредством которого контакт стабилизируется в модели в «надлежащим» образом поляризованном случае, как можно видеть на рис. 2. Более того, в пространстве параметров существует область, в которой как «правильно», так и «неправильно» поляризованные клетки стабилизируют синапсы согласно предсказаниям модели – эта область является пересечением оранжевых (стабилизационных) областей на рис. 2а и 3а и располагается вблизи левого нижнего угла построенного на графике пространства параметров (рис. 2а, 3а). Такое поведение модели, однако, не согласуется с нашими предшествующими экспериментальными измерениями, которые продемонстрировали зависимость стабильности контакта от ориентации ЦОМТ. Численный анализ ограничивает зону поведения, согласующегося с опытами, областью, которая очерчена черной линией на рис. 3а. Тот нетривиальный факт, что такая область существует, показывает, что гипотеза структурного «проверочного считывания» в ходе взаимодействия ТК и АПК количественно непротиворечива и что наблюдаемая в опытах динамика ТК поддается объяснению в рамках сравнительно простого пространственно-кинетического формализма.

**Роль рециклирования ТКР в латеральной миграции контакта ТК с АПК**

Анализируя численно общий случай произвольной ориентации рециклирования по отношению к месту инициации контакта, мы наблюдали кажущуюся миграцию расширяющегося контакта, как единого целого, в направлении точки рециклирования на клеточной поверхности. Миграция зоны контакта вокруг ТК происходила путем выдвижения границы контакта, которая уже была ближе к точке рециклирования, в то время как более удаленная от нее синаптическая граница отступала. После миграции в такой манере и накрывания точки рециклирования на ПМ, контакт был в состоянии стабилизироваться схожим образом с контактом, инициированным уже в этой ориентации. Миграция контакта вокруг клетки фактически ориентировала внутриклеточный аппарат рециклирования по отношению к зоне контакта так, как необходимо для стабильного и продуктивного взаимодействия ТК и АПК. Такое поведение, демонстрируемое моделью, указывало на то, что роль рециклирования рецепторов может быть не ограничена «проверочным считыванием» структурной полярности ТК, но что рециклирование может также играть и более непосредственную роль в происхождении такой полярности.

Модель предсказывает, что градиент плотности рецепторов, создаваемый конститутивным поляризованным рециклированием, уже существует на поверхности ТК в тот момент, когда она приходит в соприкосновение с АПК. А именно, граница начинающего развиваться контакта имеет более высокую плотность рецепторов на той стороне контакта, которая расположена ближе к РК, чем на той стороне, которая от него более удалена. Вначале, как обсуждается выше, плотность рецепторов на всех сторонах контакта превышает критическое значение для расширения. Для того, чтобы расширение превратилось в латеральную миграцию, т.е., в расширение с одной стороны и сужение с другой, плотность рецепторов на более удаленной от РК границе должна быть снижена до значений ниже критического. Это достигается в модели путем индуцированной интернализации в пределах самого контакта – снижение плотности в зоне контакта делает плотность на той границе, условия на которой уже были относительно неблагоприятны для расширения, ниже критической раньше всего. На границе, наиболее близкой к РК, как показывает численный анализ, плотность может оставаться выше критической в течение всей миграции контакта. При этом также играют роль два дополнительных фактора. Эффект собственно рециклирования (возврата на ПМ), хотя оно и задержано вследствие продолжительного времени пребывания рецепторов в РК, заключается в усилении градиента, способствующего миграции зоны взаимодействия, путем транспортировки большего числа рецепторов в область, прилегающую к РК, что означает – ближе к выдвигающейся границе контакта. Другой эффект обязан своим происхождением самой миграции. Выдвигающаяся граница перемещается в область ПМ, которая в основном не подверглась влиянию индуцированной интернализации в пределах площади контакта. Этот эффект способен к самоускорению: чем быстрее продвижение границы, тем выше плотность рецепторов на ней, поскольку ее продвижение обгоняет тогда диффузию рецепторов в обедненную область в большей степени. Отступающая же граница в то же время лишь перемещается глубже в область, обедненную рецепторами вследствие того, что она была внутренностью зоны контакта в течение длительного времени.

**Отношение модели миграции контакта к моделям градуированной агдезии**

Принимая во внимание роль передачи сигнала от ТКР к актиновому цитоскелету, который является движущей силой распространения области контакта ТК с поверхностью, связывающей ТКР [19,20,38], мы не интерпретируем формирование контакта в нашей модели исключительно как следствие опосредованной рецепторами адгезии. Латеральная миграция зоны контакта, таким образом, не может быть приписана исключительно тому обстоятельству, что адгезия сильнее там, где плотность ТКР выше. Тем не менее, связывание клеточной поверхности на одной стороне зоны контакта и открепление на противоположной ее стороне в нашей модели очень сильно напоминает механизм градуированной адгезии в моделях клеточной локомоции. Поляризованный внутриклеточный транспорт рециклируемых рецепторов моделировался в качестве механизма, который мог бы создавать градиент такого рода рецепторов на поверхности подвижной клетки и таким образом вносить вклад в, быть ответственным за, или направлять движение клетки по поверхности, к которой данные рецепторы обладают сродством [15–18]. Миграция достигается прикреплением на стороне зоны контакта, куда направлено рециклирование, и откреплением на противоположной стороне. По сравнению с этой ситуацией, наша модель применима к клеткам, которые не являются плоскими, а остаются в целом близкими к шарообразным в контакте со связывающим ТКР субстратом. Еще более важно то, что мы не предполагаем, что полярность рециклирования фиксирована в лабораторной системе координат. Поляризованное рециклирование в данной ситуации не обязательно направлено к границе контакта. Оно направлено к точке на поверхности, положение которой относительно области контакта может меняться благодаря динамике контакта. Для того, чтобы подчеркнуть отличие нашей модели от предшествующих моделей градуированной адгезии, рассмотрим ситуацию, которая возможна лишь в нашей модели: такую, в которой точка рециклирования расположена исходно за пределами площади контакта, хотя и недалеко от его границы. Адгезия тогда благоприятствуется на ближайшей границе контакта таким же образом, как в предшествующих моделях градуированной адгезии. Однако по мере того, как адгезия развивается в граничной точке возле точки рециклирования, включение клеточной поверхности в состав контакта клетки с субстратом поглощает свободную клеточную поверхность, и это сокращает расстояние вдоль поверхности клетки между контактом и точкой, к которой рециклируют рецепторы. В итоге это ведет в нашей модели к включению области рециклирования в область контакта. После того, как границы контакта охватывают точку рециклирования, рецепторы распределяются к ним поровну. Обе границы с этого момента адгезивны в равной мере, вследствие чего точка рециклирования не в состоянии когда-либо в дальнейшем покинуть площадь контакта. С одной стороны, это делает нашу модель неприменимой к непрерывной клеточной локомоции, на описание которой были направлены предыдущие модели градуированной адгезии [15–18]. С другой стороны, это представляет собой совершенно новую гипотезу о механизме ориентации рециклирования ТКР к области контакта ТК и АПК, которая наблюдается в опытах [3].

**Отношение модели миграции контакта к моделям транслокации ЦОМТ**

Фактическая миграция зоны контакта между ТК и АПК латерально вокруг ТК к области, куда направлено рециклирование, которая демонстрировалась нашей моделью, была полной противоположностью нашего исходного представления о внутриклеточной миграции РК, как части комплекса РК, ЦОМТ и АГ, к области контакта [3–10]. Будучи моделью поверхностного распределения рецепторов и динамики опосредованного рецепторами контакта, наша модель не разрабатывалась с целью предсказания взаимной ориентации зоны контакта и полярности внутриклеточного рециклирования. Наоборот, определенная ориентация РК и контакта предполагалась частью начальных условий и имплицитно ожидалось, что она должна оставаться неизменной. То, что модель тем не менее демонстрировала спонтанную переориентацию опосредованного рецепторами контакта ТК и АПК по отношению к поляризованному рециклированию рецепторов внутри ТК, заслуживает отдельного обсуждения.

Движущая сила наблюдаемого в опытах совпадения ориентации комплекса РК, ЦОМТ и АГ с контактом между ТК и АПК не выяснена. Ведущая гипотеза постулирует притяжение между ЦОМТ и зоной контакта, генерируемое кортикальными молекулярными моторами и опосредованное микротрубочками [39,40], чья сборка и разборка также может быть вовлечена в перемещение ЦОМТ [41,42]. Основанный на притяжении механизм особенно хорошо согласовывался бы с относительным движением ЦОМТ и контакта, которое было бы «векториальным», т.е. при котором ЦОМТ двигался бы к области контакта, пересекая внутренность ТК. Такого рода векториальное перемещение было документировано с использованием уникального поляризационного микроскопа, дававшего двумерное, живое изображение экспериментальной системы, включавшей ТК и АПК, которые обе были частично иммобилизированы на субстрате [39].

В сравнении с механизмом, основанным на притяжении, миграция зоны контакта вокруг ТК к области, ближайшей к РК в нашей модели предполагает прежде всего, что внутриклеточный РК изначально расположен эксцентрично. Эксцентричное и по существу подмембранное положение комплекса РК, ЦОМТ и АГ в ТК независимо от его ориентации по отношению к синапсу документировано многочисленными данными, включая наши собственные [3,5,9,10]. Более специфическим требованием достижения взаимосогласованной ориентации комплекса РК, ЦОМТ и АГ с областью контакта путем миграции последней по поверхности ТК является то, что если контакт зафиксирован на связывающей ТКР поверхности, то должно наблюдаться согласованное вращательное движение всей ТК по отношению к контакту. Мы документируем такое согласованное движение в нашей экспериментальной системе, которая заменяет АПК искусственным, неподвижным связывающим ТКР субстратом. Трехмерные изображения ТК с дифференциально окрашенными АГ и ядром показывают, что эти две органеллы, вместе составляющие бòльшую часть объема ТК, двигаются как единое составное тело по отношению к иммобилизованной площади контакта. Это наблюдение поэтому может быть эквивалентно описано как то, что площадь контакта движется вокруг ТК таким же образом, как это происходит в модели.

Тем не менее, строго говоря, какой бы то ни было механизм, основанный на притяжении, требует лишь относительного движения контакта и комплекса РК, ЦОМТ, и АГ, поскольку эти две структуры подвергаются согласно этой гипотезе действию и противодействию. Векториальная транслокация комплекса РК, ЦОМТ и АГ через внутренность ТК не является, строго говоря, необходимой – его движение по дуге под поверхностью ТК также было бы совместимо с тем, что он притягивается к области контакта если, как представляется, особенно массивное ядро ТК преграждает путь через центральную часть клетки. Таким образом, несмотря на то, что характер относительного движения контакта и АГ, которое наблюдается в наших трехмерных и обладающих достаточным временным разрешением данных, совместим с миграцией контакта к АГ, эти данные не позволяют заключить, движется ли активно контакт к АГ или АГ к контакту. Этот вопрос невозможно разрешить посредством наблюдения относительного движения этих двух структур.

В поисках дополнительных черт динамики ТК, необходимых для ответа на поставленный выше вопрос, мы обратили внимание на форму, а не только положение контакта между ТК и субстратом. Наблюдалось асимметричное расширение контакта: более выраженное на стороне, которая была ближе к АГ. Асимметрия расширения контакта указывает на возможность, что расширение контакта является движущей силой относительного движения контакта и комплекса РК, ЦОМТ и АГ. Этот довод основан на сравнении полноты двух возможных объяснений опытов. Относительное движение контакта и комплекса РК, ЦОМТ и АГ в самом деле может быть одним и тем же независимо от того, где приложена движущая сила. Однако, если эта сила приводит комплекс РК, ЦОМТ и АГ в движение через цитоплазму, то асимметрия расширения контакта между ТК и субстратом остается необъясненной. Если же, наоборот, движущей силой является расширение контакта, то тогда как относительное движение, так и асимметрия экспансии оба объясняются. Мы заключаем, что в значительной мере имеет место движение контакта между ТК и АПК к комплексу РК, ЦОМТ и АГ, а не комплекса – к контакту.

Резюмируя, предложенная модель обобщает предшествующие модели рециклирования ТКР путем введения нового уровня деталей и динамики для описания реалистичного взаимодействия ТК с АПК. Это позволило нам объяснить количественно проведенные ранее эксперименты, сформулировав количественную теорию структурного проверочного считывания в ходе взаимодействия ТК и АПК. Наконец, обобщенная модель предсказала новый механизм, вносящий вклад в поляризацию ТК в целом: латеральную миграцию области контакта ТК и АПК, которая позиционирует межклеточный контакт по отношению к аппарату рециклирования рецепторов в ТК. Предсказание было подкреплено новыми опытами.

МАТЕРИАЛЫ И МЕТОДЫ

**Математическое моделирование**

Модель описывается следующими уравнениями:

$\frac{\partial P\left(\varphi,t\right)}{\partial t}=D\frac{\partial^{2}P\left(\varphi,t\right)}{\partial\varphi^{2}}-k\left(\varphi,t\right)P\left(\varphi,t\right)+\delta\left(\varphi-\varphi_{r}\right)k_{r}r\left(t\right)$,

$k\left(\varphi,t\right)=\left\{\begin{matrix} k_{c}, \varphi<\varphi_{1}\left(t\right) \\ k_{i}, \varphi_{1}\left(t\right)\leq\varphi\leq\varphi_{2}\left(t\right) \\ k_{c}, \varphi>\varphi_{2}\left(t\right) \end{matrix}\right.$,

$\frac{dr\left(t\right)}{dt}=\int_{0}^{2\pi} k\left(\varphi,t\right)P\left(\varphi,t\right)d\varphi-k_{r}r\left(t\right)$,

$\frac{d\varphi_{1}\left(t\right)}{dt}=-k_{\omega}\left(P\left(\varphi_{1},t\right)-p_{\text{крит}}\right)$,

$\frac{d\varphi_{2}\left(t\right)}{dt}=k_{\omega}\left(P\left(\varphi_{2},t\right)-p_{\text{крит}}\right)$.

Здесь, *δ* – дельта-функция Дирака. Граничные условия для *P* на *φ =*0, 360° – периодические. Начальные условия *P*(*φ*, 0), *r*(0) являются стационарным решением модификации вышеприведенной модели, в которой движущиеся границы не принимаются во внимание и *k* полагается повюду равной *k*_c_. Таким образом, начальные условия для расчета динамики, следующей за соприкосновением ТК и АПК, представляют собой стационарное распределение рецепторов, которое достигается в ТК в отсутствие сигнала перед тем, как она входит в контакт с АПК. Начальное условие *φ*_1,2_(0) = 270° обозначает инициацию формирования контакта внизу ТК, помещая нашу экспериментальную ситуацию (см. ниже) в общепринятую полярную систему координат модели (рис. 1). В случае, если *φ*_1_(*t*) = *φ*_2_(*t*) в любой момент *t*>0, симуляция прекращается и за ее результат принимается коллапс контакта. В случае, если *φ*_2_(*t*)–*φ*_1_(*t*) > 180°, симуляция также прекращается и ее результатом считается нереалистично большое расширение контакта. Вышеприведенная математическая формулировка упрощается тем обстоятельством, что в описанных симуляциях как *φ*_1_, так и *φ*_2_ остаются в пределах 0 и 360°.

Мы используем следующие значения констант скорости, которые были измерены в ТК линии Юркат: *k*_c_ = 0,012 мин^–1^, *k*_i_ = 0,128 мин^–1^, *k*_r_ = 0,055 мин^–1^ [2,4]. Принимая во внимание коэффициент диффузии поверхностного ТКР в ТК линии Юркат (0,12 мкм^2^/с [11]) и приблизительный радиус (7,5 мкм) используемых в опытах ТК этой линии [5], угловая диффузионная константа может быть рассчитана как *D* = (0,12 мкм^2^/с)/(7,5 мкм/рад)^2^ = 0,128 рад^2^/мин. Константа скорости границы синапса *k*_ω_ и критическая плотность рецепторов *p*_крит_, требуемая для локальной экспансии синапса, варьируются при анализе модели. Модель была дискретизирована с однородным Δ*φ* и решена прямым методом Эйлера в программе МАТЛАБ.

**Экспериментальные процедуры**

Клетки линии Юркат культивировались и подготавливались к наблюдению в основном так, как было описано ранее [5,38]. Вкратце, клетки, суспендированные в культуральной среде RPMI1640 (Invitrogen), инъецировались в камеру для наблюдений (LabTek). Дно камеры было стеклянным и покрытым антителами к ТКР (клон UCHT1). Осаждающиеся клетки наблюдались на инвертированном микроскопе (Nikon TE-200) с использованием охлаждаемой ПЗС-камеры (Hamamatsu ORCA-II). Водно-иммерсионный планапохроматический объектив с численной апертурой 1,2 (Nikon) приводился в движение пьезо-электрическим устройством (Physik Instrumente PIFOC-721). Камера, привод объектива, и затвор (Uniblitz) контролировались программой IPLab, которая использовалась также для анализа изображения. Температура (37 °C) поддерживалась инкубатором воздушного потока (ASI-400). Путем перемещения объектива регистрировались трехмерные цифровые изображения с формальным разрешением (размером воксела) 0,22, 0,22 и 0,4 мкм соответственно вдоль осей X, Y и Z, из которых Z была направлена вдоль оптической оси и ортогональна стеклу, образующему дно камеры для наблюдений.

Для изучения движений АГ и ядра эти две органеллы были помечены соответственно C5-церамидом, конъюгированным с флуорофором BODIPY-FL и Хехстом 33342 (Molecular Probes) перед впрыскиванием в камеру для наблюдений. С этой целью после предварительной инкубации с меченым церамидом (с добавлением бычьего сывороточного альбумина) в концентрации 5 мкмоль/л в течение 10 мин, добавлялся Хехст 33342 до концентрации 1мкг/мл и клетки инкубировались с обеими метками в течение еще 20 мин при 37 ºC и 5% CO_2_. Изображения регистрировались с использованием конфокальной приставки на основе диска Нипкова (CARV-II). На каждой временной точке, стеки изображений регистрировались отдельно на длинах волн меток АГ и ядра. Регистрация каждого Z-стека занимала 7,5 с.

Для изучения взаимной ориентации АГ и первоначального вытягивания контакта, клетки предварительно инкубировались с флуоресцентной меткой АГ брефельдином-BODIPY558 (Molecular Probes) в концентрации 0,1 мкмоль/л в течение 20 мин. Трехмерные изображения регистрировались по отдельности на длине волны, соответствующей метке АГ и в проходящем свете, показывающем зону контакта клетки с субстратом.

СПИСОК ЦИТИРОВАННОЙ ЛИТЕРАТУРЫ

1. Alberts B, Roberts K, Lewis J, Raff M, Bray D (1989) Molecular biology of the cell. New York: Garland. 1219 p.
2. Geisler C (2004) TCR trafficking in resting and stimulated T cells. Crit Rev Immunol 24: 67–86.
3. Das V, Nal B, Dujeancourt A, Thoulouze M-I, Galli T, Roux P, Dautry-Varsat A, Alcover A (2004) Activation-induced polarized recycling targets T cell antigen receptors to the immunological synapse: involvement of SNARE complexes. Immunity 20: 577–588.
4. Menne, C, Sorensen T, Siersma V, von Essen M, Odum N, Geisler C (2002) Endo- and exocytic rate constants for spontaneous and protein kinase C-activated T cell receptor cycling. Eur J Immunol 32: 616–626.
5. Arkhipov SN, Maly IV (2006) Quantitative analysis of the role of receptor recycling in T cell polarization. Biophys J 91: 4306–4316.
6. Wülfing C, Davis MM (1998) A receptor/cytoskeletal movement triggered by costimulation during T cell activation. Science 282: 2266–2269.
7. Moss WC, Irvine DJ, Davis MM, Krummel MF (2002) Quantifying signaling-induced reorientation of T cell receptors during immunological synapse formation. Proc Natl Acad Sci U S A 99: 15024–15029.
8. Burkhardt JK, Echeverri CJ, Nilsson T, Vallee RB (1997) Overexpression of the dynamitin (p50) subunit of the dynactin complex disrupts dynein-dependent maintenance of membrane organelle distribution. J Cell Biol 139: 469–484.
9. Geiger B, Rosen D, Berke G (1982) Spatial relationships of microtubule-organizing centers and the contact area of cytotoxic T lymphocytes and target cells. J Cell Biol 95: 137–143.
10. Kupfer A, Singer SJ (1989) Cell biology of cytotoxic and helper T cell functions: immunofluorescence microscopic studies of single cells and cell couples. Annu Rev Immunol 7: 309–337.
11. Favier B, Burroughs NJ, Wedderburn L, Valitutti S (2001) TCR dynamics on the surface of living T cells. Int Immunol 13: 1525–1532.
12. Kuhne MR, Lin J, Yablonski D, Mollenauer MN, Ehrlich LIR, Huppa J, Davis MM, Weiss A (2003) Linker for activation of T cells, ζ-associated protein-70, and Src homology 2 domain-containing leukocyte protein-76 are required for TCR-induced microtubule-organizing center polarization. J Immunol 171: 860–866.
13. Kupfer A, Dennert G, Singer SJ (1985) The reorientation of the Golgi apparatus and the microtubule-organizing center in the cytotoxic effector cell is a prerequisite in the lysis of bound target cells. J Mol Cell Immunol 2: 37–49.
14. Hammer DA, Lauffenburger DA (1987) A dynamical model for receptor-mediated cell adhesion to surfaces. Biophys J 52: 475–487.
15. DiMilla PA, Barbee K, Lauffenburger DA (1991) Mathematical model for the effects of adhesion and mechanics on cell migration speed. Biophys J 60: 15–37.
16. Lauffenburger DA, Linderman JJ (1993) Receptors: Models for binding, trafficking, and signaling. Oxford: Oxford University Press. 365 p.
17. Dunn GA, Zicha D (1995) Dynamics of fibroblast spreading. J Cell Sci 108: 1239–1249.
18. Mogilner A, Marland E, Bottino D (2001) A minimal model of locomotion applied to the steady 'gliding' movement of fish keratocyte cells. In: Othmer H, Maini P, editors. Pattern formation and morphogenesis: Basic processes. New York: Springer. pp. 269–294
19. Parsey MV, Lewis GK (1993) Actin polymerization and pseudopod reorganization accompany anti-CD3 induced growth arrest in Jurkat T cells. J Immunol 151: 1881–1893.
20. Borroto A, Gil D, Delgado P, Vicente-Manzanares M, Alcover A, Sanchez-Madrid F, Alarcon B (2000) Rho regulates T cell receptor ITAM-induced lymphocyte spreading in an integrin-independent manner. Eur J Immunol 30: 3403–3410.
21. Bunnell SC, Kapoor V, Trible RP, Zhang W, Samelson LE (2001) Dynamic actin polymerization drives T cell receptor-induced spreading: A role for the signal transduction adaptor LAT. Immunity 14: 315–329.
22. Bray D (2001) Cell Movements: From molecules to motility. New York: Garland. 372 p.
23. Monks CR, Freiberg BA, Kupfer H, Sciaky N, Kupfer A (1998) Three-dimensional segregation of supramolecular activation clusters in T cells. Nature 395: 82–86.
24. Qi SY, Groves JT, Chakraborty AK (2001) Synaptic pattern formation during cellular recognition. Proc Natl Acad Sci U S A 98: 6548–6553.
25. Burroughs NJ, Wülfing C (2002) Differential segregation in a cell-cell contact interface: the dynamics of the immunological synapse. Biophys J 83: 1784–1796.
26. Sancho D, Vicente-Manzanares M, Mittelbrunn M, Montoya MC, Gordon-Alonso M, Serrador JM, Sanchez-Madrid F (2002) Regulation of microtubule-organizing center orientation and actomyosin cytoskeleton rearrangement during immune interactions. Immunol Rev 189: 84–97.
27. Coombs D, Kalergis AM, Nathenson SG, Wofsy C, Goldstein B (2002) Activated TCRs remain marked for internalization after dissociation from pMHC. Nat Immunol 3: 926–931.
28. Wofsy C, Coombs D, Goldstein B (2001) Calculations show substantial serial engagement of T cell receptors. Biophys J 80: 606–612.
29. Gakamsky DM, Luescher IF, Pramanik A, Kopito RB, Lemonnier F, Vogel H, Rigler R, Pecht I (2005) CD8 kinetically promotes ligand binding to the T-cell antigen receptor. Biophys J 89: 2121–2133.
30. Gonzalez PA, Carreno LJ, Coombs D, Mora JE, Palmieri E, Goldstein B, Nathenson SG, Kalergis AM (2005) T cell receptor binding kinetics required for T cell activation depend on the density of cognate ligand on the antigen-presenting cell. Proc Natl Acad Sci U S A 102: 4824–4829.
31. Lee K-H, Dinner AR, Tu C, Campi G, Raychaudhuri S, Varma R, Sims TN, Burack WR, Wu H, Wang J, Kanagawa O, Markiewicz M, Allen PM, Dustin ML, Chakraborty AK, Shaw AS (2003) The immunological synapse balances T cell receptor signaling and degradation. Science 302: 1218–1222.
32. Valitutti S, Müller S, Cella M, Padovan E, Lanzavecchia A (1995) Serial triggering of many T-cell receptors by a few peptide-MHC complexes. Nature 375: 148–151.
33. Reichert P, Reinhardt RL, Ingulli E, Jenkins MK (2001) In vivo identification of TCR redistribution and polarized IL-2 production by naive CD4 T cells. J Immunol 166: 4278–4281.
34. McGavern, DB, Christen U, Oldstone MBA (2002) Molecular anatomy of antigen-specific CD8+ T cell engagement and synapse formation in vivo. Nat Immunol 3: 918–925.
35. Kupfer A, Dennert G (1984) Reorientation of the microtubule-organizing center and the Golgi apparatus in cloned cytotoxic lymphocytes triggered by binding to lysable target cells. J Immunol 133: 2762–2766.
36. Cai Y, Biais N, Giannone G, Tanase M, Jiang G, Hofman JM, Wiggins CH, Silberzan P, Buguin A, Ladoux B,. Sheetz MP (2006) Nonmuscle myosin IIA-dependent force inhibits cell spreading and drives F-actin flow. Biophys J 91: 3907–3920.
37. Nicklas RB, Ward SC, Gorbsky GJ (1995) Kinetochore chemistry is sensitive to tension and may link mitotic forces to a cell cycle checkpoint. J Cell Biol 130: 929–939.
38. Kuhn JR, Poenie M (2002) Dynamic polarization of the microtubule cytoskeleton during CTL-mediated killing. Immunity 16: 111–121.
39. Combs J, Kim SJ, Tan S, Ligon LA, Holzbaur ELF, Kuhn J, Poenie M (2006) Recruitment of dynein to the Jurkat immunological synapse. Proc Natl Acad Sci U S A 103: 14883–14888.
40. Stowers L, Yelon D, Berg LJ, Chant J (1995) Regulation of the polarization of T cells toward antigen-presenting cells by Ras-related GTPase CDC42. Proc Natl Acad Sci U S A 92: 5027–5031.
41. Lowin-Kropf B, Smith Shapiro V, Weiss A (1998) Cytoskeletal polarization of T cells is regulated by an immunoreceptor tyrosine-based activation motif-dependent mechanism. J Cell Biol 140: 861–871.
42. Bunnell SC,. Barr VA, Fuller CL, Samelson LE (2003) High-resolution multicolor imaging of dynamic signaling complexes in T cells stimulated by planar substrates. Sci STKE 2003: pl8.


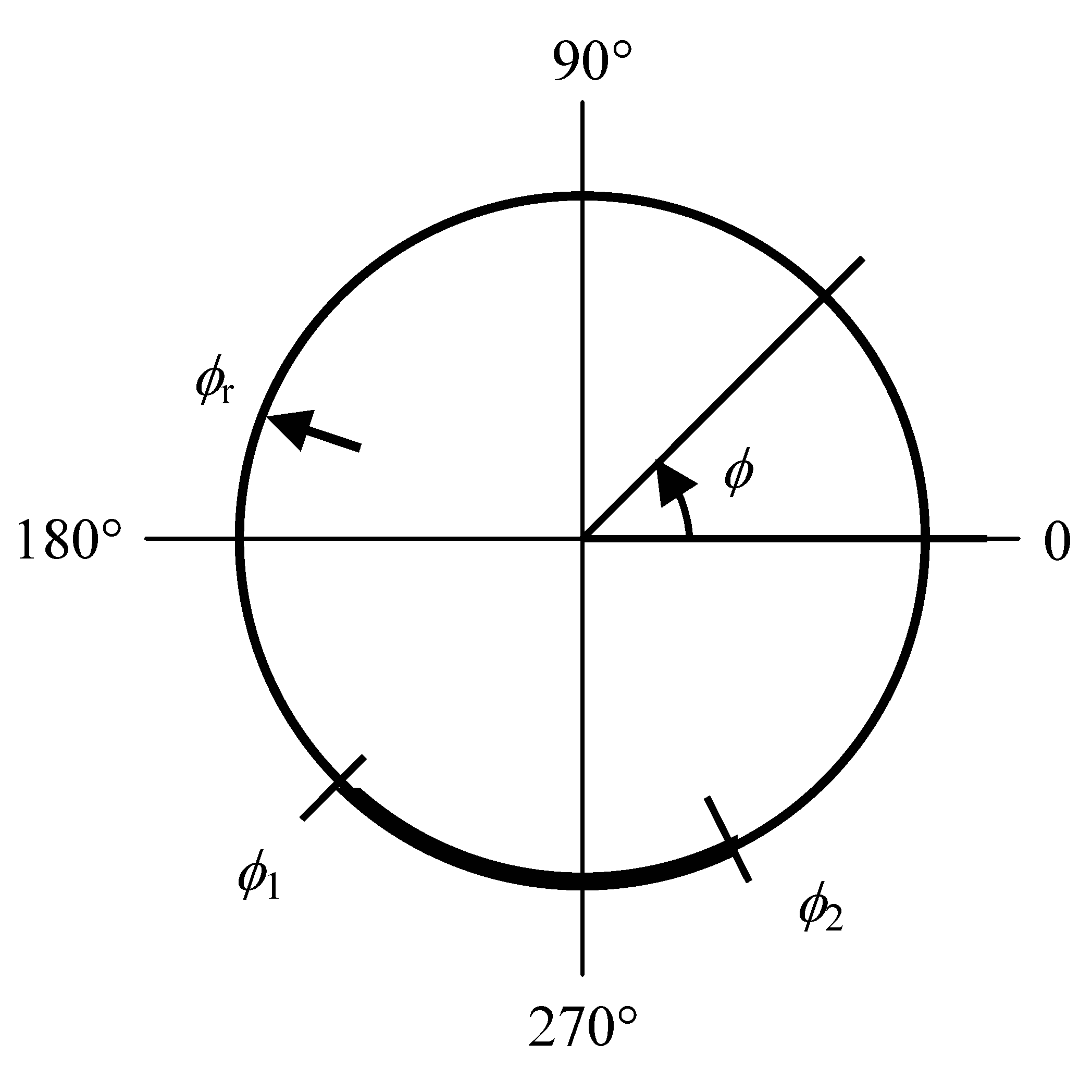


**Рис. 1. Схематическая диаграмма математической модели.** Окружность обозначает поверхность ТК, на которой ТКР распределяется путем латеральной диффузии. Положение на поверхности задается угловой координатой *φ*, как показано на рисунке. *φ*_1_ и *φ*_2_ обозначают мгновенные положения двух движущихся границ области контакта между ТК и АПК, которая показана жирной дугой. ТКР интернализуется из зоны контакта с высокой, индуцированной лигандом константой скорости *k*_i_, а из остальной части поверхности – с более низкой, конститутивной константой скорости *k*_c_. Поляризованное рециклирование интернализированных рецепторов направлено в положение, обозначенное *φ*_r_, которое прилегает к эксцентричному внутриклеточному РК, не показанному на диаграмме. Положения точки рециклирования и границ на диаграмме являются произвольными. В модели точка рециклирования *φ*_r_ зафиксирована, в то время как границы *φ*_1_ и *φ*_2_ могут перемещаться по поверхности, вызывая расширение или сокращение области контакта в соответствии с тем, является ли местная плотность рецепторов *P*(*φ*, *t*) на границах выше или ниже критической плотности *p*_крит_ для прикрепления к АПК.


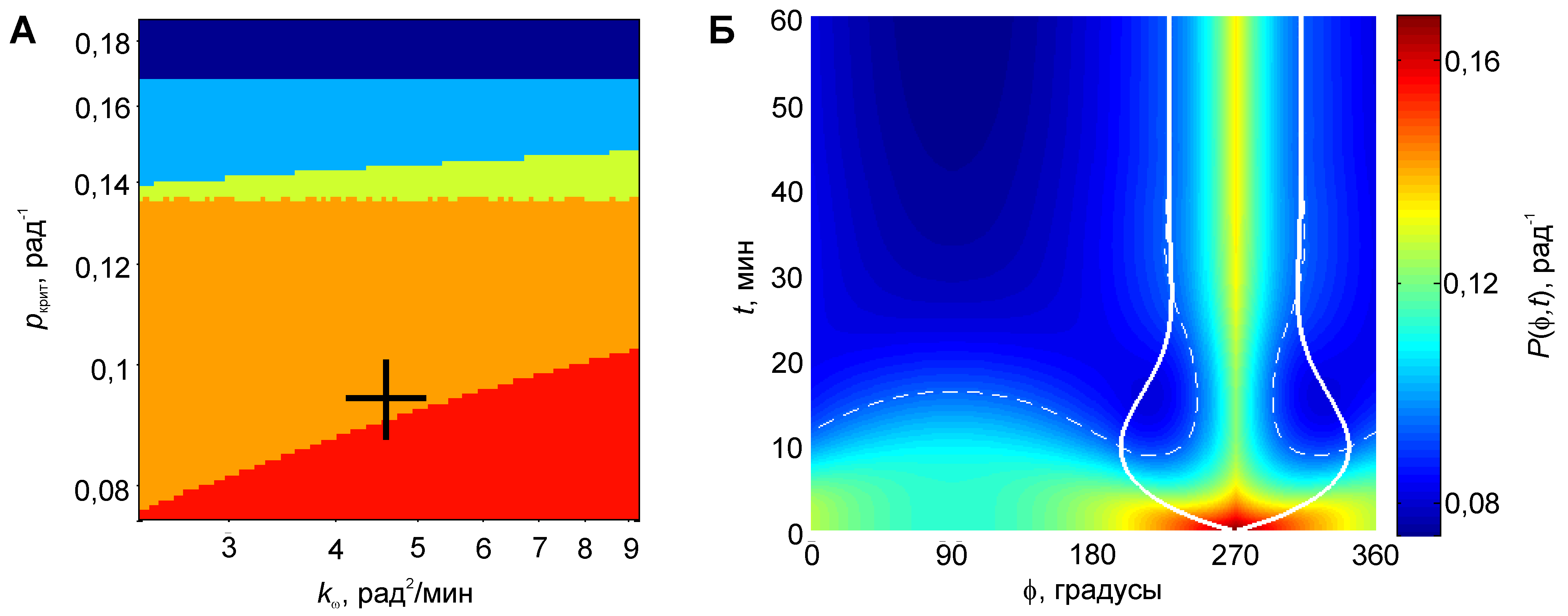


A

**Рис. 2. Поведение модели в случае, когда точка рециклирования ТКР совпадает с точкой инициации контакта с АПК.** (***A***) Области в пространстве параметров, которые задают качественно различные динамические сценарии. По осям: *p*_крит_ – критическая плотность ТКР для прикрепления к АПК; *k*_ω_ – константа скорости расширения контакта. Остальные параметры принимают экспериментально измеренные значения. *Темно-синий* – контакт не развивается; *светло-синий* – развивается стабильный контакт незначительного размера (<30° дуги); *желтый –* контакт коллапсирует неполностью после того, как кратковременно превышает 30° дуги; *оранжевый* – контакт стабилизируется выше 30° дуги; *красный* – модель предсказывает нереалистичное расширение >180°. *Крест* обозначает комбинацию параметров, используемую в динамическом примере, показанном в части *Б* настоящего рисунка: *p*_крит_ = 0,09 рад^–1^, *k*_ω_ = 4,7 рад^2^/мин. (***Б***) Динамика распределения поверхностного ТКР *P*(*φ*, *t*) и границ контакта *φ*_1,2_(*t*). Плотность ТКР *P* показана в псевдо-цвете, в единицах доли полного количества ТКР в клетке на 1 радиан клеточной окружности. Непрерывные линии показывают положение границ контакта *φ*_1_, *φ*_2_. Штриховая линия является изолинией критической плотности ТКР для расширения контакта, *P* = *p*_крит_. *φ*  – угловая координата, указывающая положение вокруг ТК, причем 270° соответствует низу клетки, куда предполагается направленным рециклирование и где в наших опытах инициируется контакт с субстратом, связывающим ТКР. *t* – время после инициации контакта (так что горизонтальная линия, проведенная через график, соответствует мгновенным распределению ТКР и положениям границ контакта в момент времени, соответствующий высоте, на которой проведена эта воображаемая линия).


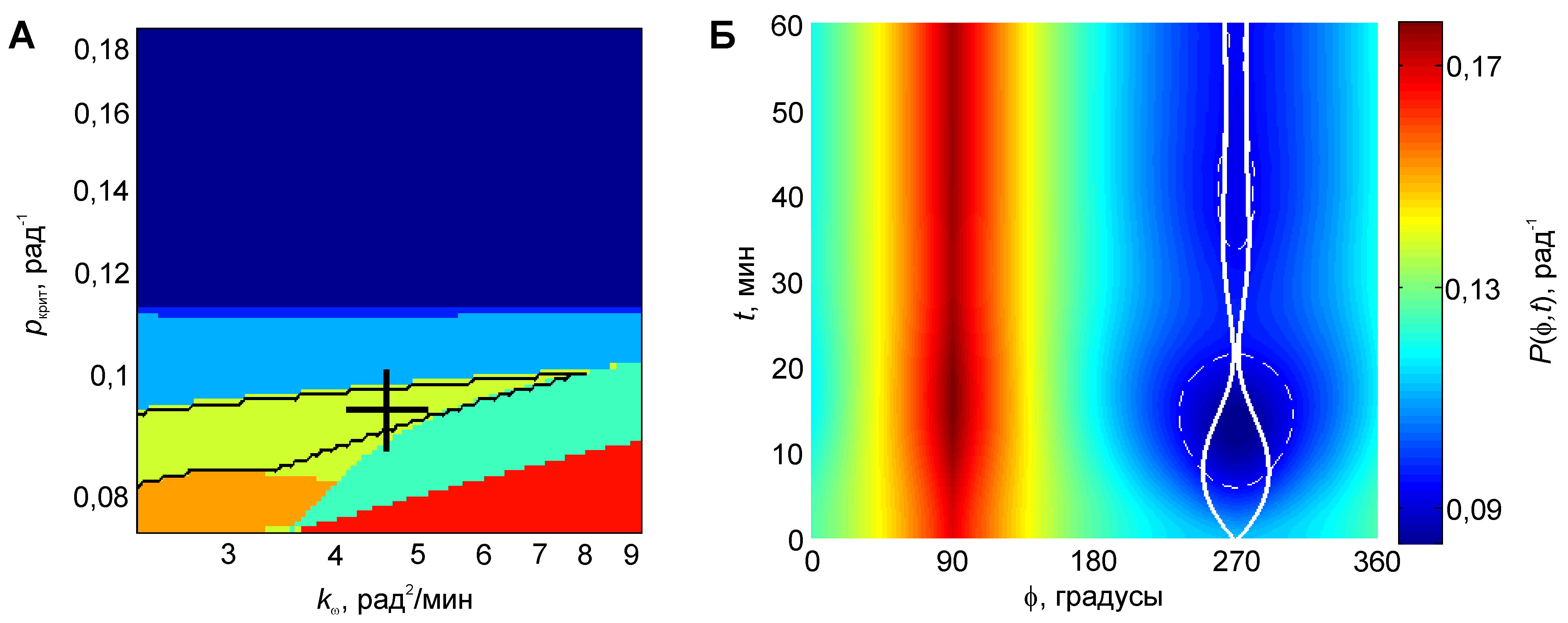


**Рис. 3. Поведение модели в случае, когда точка рециклирования ТКР противолежит точке инициации контакта с АПК.** (***A***) Области в пространстве параметров, которые задают качественно различные динамические сценарии. Цветовая кодировка та же, что на рис. 2а, с двумя дополнительными режимами, возможными в данном случае: *промежуточный синий* (узкая граничная область, разделяющая темно- и светло-синюю) – контакт коллапсирует полностью, никогда не превышая 30° дуги; *бирюзовый* – контакт коллапсирует полностью после того, как он временно превышает 30° дуги. Область, *очерченная черным*, определена как пересечение областей на этом рисунке и на рис. 2а: внутри черной границы модель предсказывает, что формирование контакта значительного размера (>30°) является стабильным, если точка инициации контакта совпадает с точкой рециклирования, и лишь временным, если эти две точки диаметрально противоположны. Таким образом, внутри черной границы, модель воспроизводит предшествующие опыты. *Крест* обозначает комбинацию параметров, используемую в динамическом примере, показанном в части *Б* настоящего рисунка, которая является той же самой, что в примере на рис. 2б. (***Б***) Динамика распределения поверхностного ТКР и границ контакта. Условности те же, что на рис. 2б. Рециклирование направлено к *φ* = 90° (верх клетки в опытах), контакт инициируется в *φ* = 270° (низ клетки).


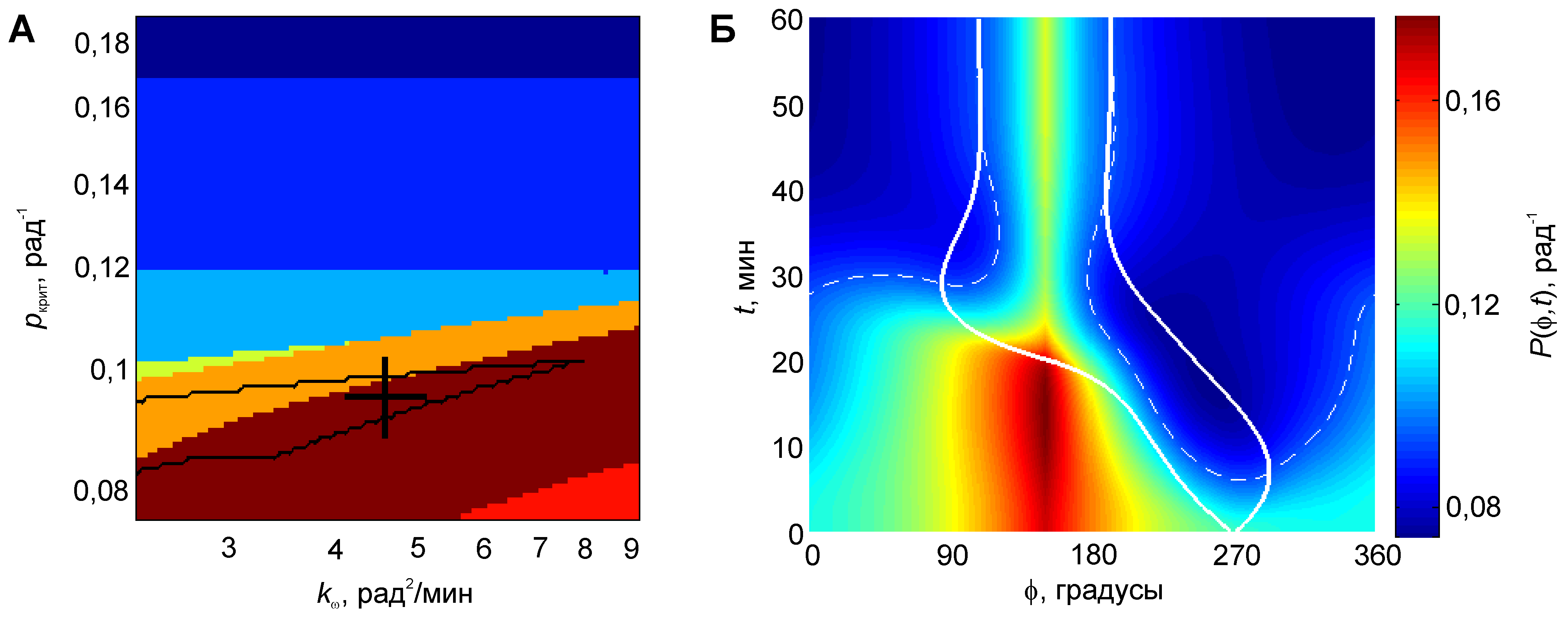


**Рис. 4. Поведение модели в случае, когда точка рециклирования отстоит от точки инициации контакта на 120°.** (***A***) Области в пространстве параметров, который задают качественно различные динамические сценарии. Цветовая кодировка та же, что на рис. 2а и 3а, с дополнительным режимом, показанным *коричневым*: в этом новом режиме, зона контакта не только стабилизируется на >30° в размере, но также перекрывает точку рециклирования не позднее, чем через 30 мин. Область, *очерченная черным*, та же, что на рис. 3а, и обозначает комбинации значений параметров, которые предсказывали поведение модели, совместимое с нашими предыдущими экспериментами. Примечательным образом, область, в которой предсказывается новый тип поведения (*коричневый*), перекрывается с областью, которая совместима с предыдущими экспериментами (*очерчено черным*). *Крест* обозначает комбинацию параметров, используемую в динамическом примере, показанном в части *Б* настоящего рисунка, которая является той же самой, что в примерах на рис. 2б и 3б. (***Б***) Динамика распределения поверхностного ТКР и границ контакта. Условности те же, что на рис. 2б и 3б. Рециклирование направлено к *φ* = 150°, контакт инициируется в *φ* = 270°, каковые положения отделены 120° вдоль окружности ТК.


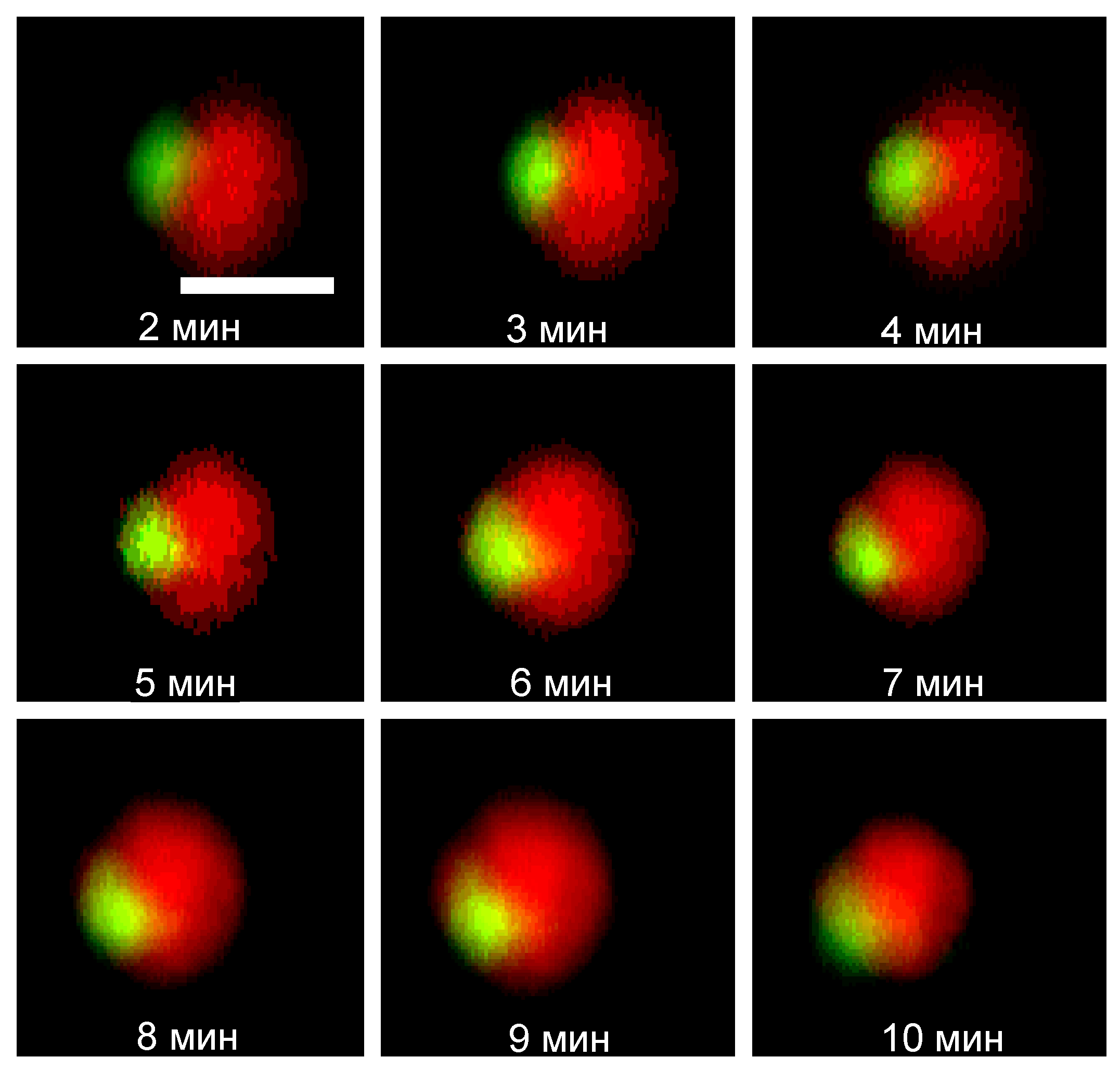


**Рис. 5. Переориентация контакта ТК с субстратом в направлении АГ.** АГ флуоресцентно помечен зеленым и ядро – красным. Клетка находится на горизонтальном, не флуоресцирующем, связывающем ТКР субстрате. Показана временная последовательность видов сбоку на трехмерные конфокальные изображения. Уровень нефлуоресцентного субстрата под клеткой примерно указан масштабным отрезком (10 мкм) на первом изображении. Можно наблюдать, как АГ приближается к субстрату по дуге. Поскольку субстрат в условиях данного эксперимента неподвижен, движение такого рода совместимо с миграцией области контакта клетки с субстратом по поверхности клетки в направлении эксцентрично расположенного АГ.


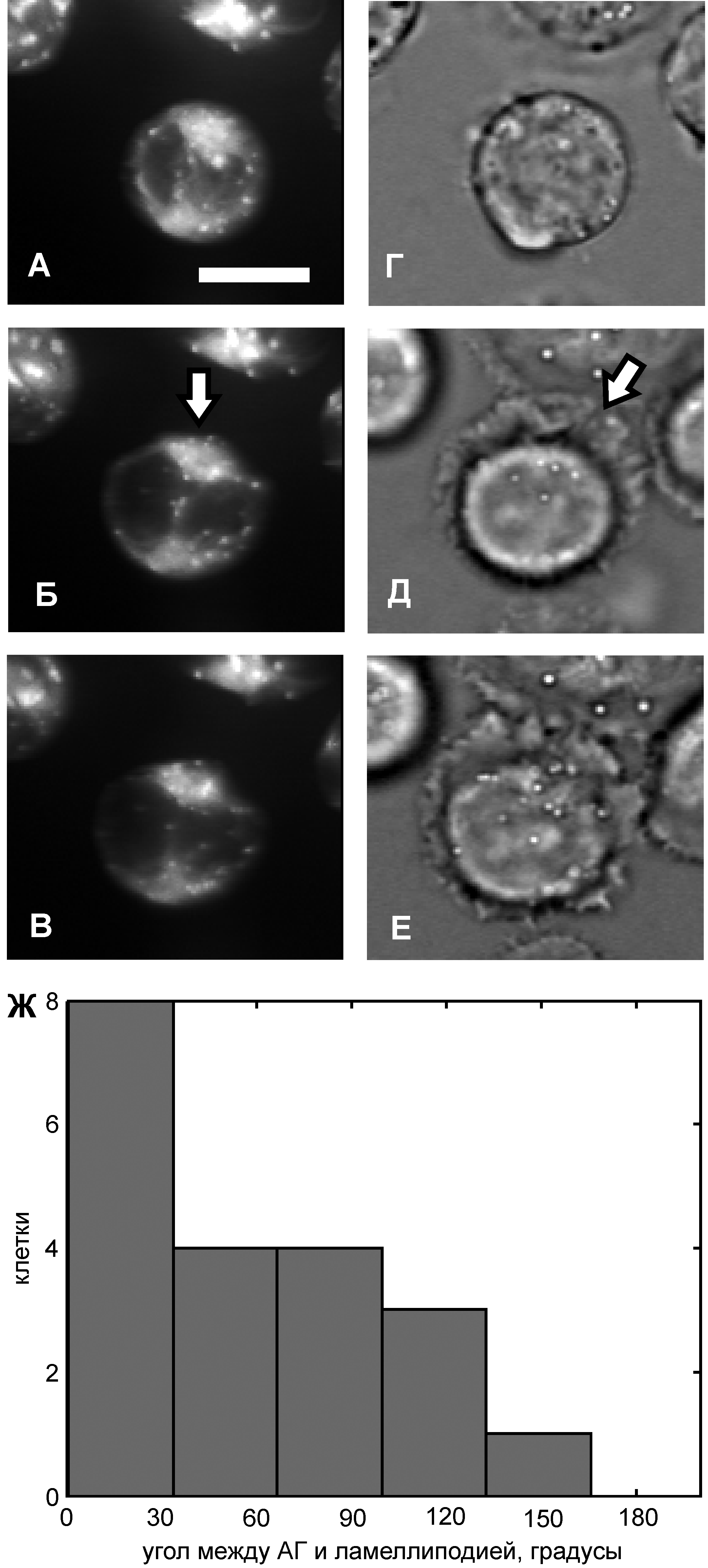


**Рис. 6. Предпочтительное вытягивание стороны контакта ТК с субстратом, которая расположена наиболее близко к АГ.** (***A-В***) Временная последовательность видов сверху на трехмерные изображения флуоресцентно меченого АГ (*стрелка* в *Б*) в ТК на связывающем ТКР субстрате. Масштабный отрезок – 10 мкм. Временной интервал – 1 мин. (***Г-Е***) Изображения в проходящем свете той же клетки на тех же временных точках. Медиана асимметрично вытянутого контакта клетки с субстратом (ламеллиподии) указана *стрелкой* в *Д*. Обратите внимание, что контакт вытягивается наиболее сильно на той его стороне, которая наиболее близка к АГ, и что его асимметрия поддается однозначному определению в начале расширения (*Д*). (***Ж***) Гистограмма углов, при виде сверху, между направлением из центроида клетки к центроиду АГ и направлением из центроида клетки к медиане ламеллиподии.
